# RELA is required for CD271 expression and stem-like characteristics in hypopharyngeal cancer

**DOI:** 10.1038/s41598-022-22736-6

**Published:** 2022-10-22

**Authors:** Akira Nakazato, Mai Mochizuki, Rie Shibuya-Takahashi, Haruna Fujimori, Keitaro Fujii, Satoshi Saijoh, Shinkichi Morita, Tomoko Yamazaki, Takayuki Imai, Ikuro Sato, Kennichi Satoh, Kazunori Yamaguchi, Kazuo Sugamura, Jun Yasuda, Kazuto Matsuura, Hideo Shojaku, Yukinori Asada, Keiichi Tamai

**Affiliations:** 1 Division of Cancer Stem Cell, Miyagi Cancer Center Research Institute, 47-1, Medeshima-Shiote, Natori, Miyagi Japan; 2 Division of Molecular and Cellular Oncology, Miyagi Cancer Center Research Institute, 47-1, Medeshima-Shiote, Natori, Miyagi Japan; 3 Department of Head and Neck Surgery, Miyagi Cancer Center, 47-1, Medeshima-Shiote, Natori, Miyagi Japan; 4 Department of Head and Neck Medical Oncology, Miyagi Cancer Center, 47-1, Medeshima-Shiote, Natori, Miyagi Japan; 5 Department of Pathology, Miyagi Cancer Center, 47-1, Medeshima-Shiote, Natori, Miyagi Japan; 6 Division of Gastroenterology, Tohoku Medical and Pharmaceutical University, 1-15-1 Fukumuro, Miyaginoku, Sendai, Miyagi 983-8536 Japan; 7 Department of Head and Neck Surgery, National Cancer Center Hospital East, Kashiwanoha, Kashiwa, Chiba Japan; 8 Department of Otolaryngology, University of Toyama, Sugitani, Toyama Japan

**Keywords:** Cancer, Cancer stem cells, Head and neck cancer

## Abstract

CD271 (also referred to as nerve growth factor receptor or p75^NTR^) is expressed on cancer stem cells in hypopharyngeal cancer (HPC) and regulates cell proliferation. Because elevated expression of CD271 increases cancer malignancy and correlates with poor prognosis, CD271 could be a promising therapeutic target; however, little is known about the induction of *CD271* expression and especially its promoter activity. In this study, we screened transcription factors and found that RELA (p65), a subunit of nuclear factor kappaB (NF-κB), is critical for *CD271* transcription in cancer cells. Specifically, we found that RELA promoted *CD271* transcription in squamous cell carcinoma cell lines but not in normal epithelium and neuroblastoma cell lines. Within the *CD271* promoter sequence, region + 957 to + 1138 was important for RELA binding, and cells harboring deletions in proximity to the + 1045 region decreased *CD271* expression and sphere-formation activity. Additionally, we found that clinical tissue samples showing elevated *CD271* expression were enriched in RELA-binding sites and that HPC tissues showed elevated levels of both CD271 and phosphorylated RELA. These data suggested that RELA increases *CD271* expression and that inhibition of RELA binding to the *CD271* promoter could be an effective therapeutic target.

## Introduction

CD271, also referred to as nerve growth factor receptor, functions at the molecular nexus of cell death, survival, and differentiation^1^. In addition to its contribution to the nervous system, recent studies have revealed that CD271 plays a role in cancer. In melanoma cells, CD271 is identified as a cancer stem cell marker^2^, but recent study suggested that high CD271 expression reduce tumor growth and metastasis^3^. In gastric cancer CD271 inhibits invasion and metastasis by suppressing NFkB signaling^4^. Meanwhile, CD271 expression is positively correlated with malignancy in squamous cell carcinoma, including, lung squamous cell carcinoma^5^, esophageal cancer^6^, and hypopharyngeal cancer (HPC)^7^. In HPC, elevated expression of CD271 correlates with poor prognosis, as well as high tumorigenicity and invasion capability^7,8^. Furthermore, CD271 expression has been identified at the invasive front of cancer cell^7^. These data suggest that regulation of CD271 expression might represent a therapeutic target.

The mechanisms of CD271 transcription have not been fully elucidated, and few studies have reported on the transcription factors involved in *CD271* promoter activity. In neuroblastoma, transcription factor specificity protein 1 (Sp1) is required for *CD271* transcription following its binding near the transcription start site^9^. Additionally, in proximal tubular renal cells, the − 41 to + 100 region of the *CD271* promoter is required for rapamycin-induced *CD271* transcription^10^. However, there are no reports of transcriptional regulation of *CD271* in head and neck squamous cell carcinoma (HNSC).

HPC is associated with the hypopharynx and accounts for 21.4% of all subsites related to HNSC in Japan^11^. The location of the hypopharynx results in the worst prognosis of HNSC among all subsites. Despite advances in chemotherapy, radiation, and reconstructive surgery options, there exists no clearly preferred treatment modality, and efforts to improve survival have been challenging and of limited efficacy^12^. Therefore, identification of a new therapeutic target is required.

In this study, we identified the promoter region and a responsible transcription factor of *CD271* in HPC and investigated *CD271*-transcription-dependent effects on tumor malignancy.

## Materials and methods

### Ethics statements

This study was conducted in accordance with the Declaration of Helsinki and approved by the Ethics Committees of the Miyagi Cancer Center (Natori, Japan). All procedures were approved by and executed in accordance with the Miyagi Cancer Center (permit No. 2018-010) and performed according to committee regulations. All patients provided written informed consent for inclusion in the study.

### Cell lines

We used HPC patient-derived xenograft cell lines (HPCM1^7,8^ and HPCM2^7,8^ cells), which were maintained in Roswell Park Memorial Institute (RPMI)-1640 medium (Wako, Osaka, Japan) supplemented with 10% fetal bovine serum (FBS), 100 unit/mL penicillin, and 100 μg/mL streptomycin. MCC148c cells^13^, established by patient-derived xenografts of cancer tissue from a lung squamous cell carcinoma patient^5^, were maintained in DMEM supplemented with 10% FBS, 0.4 mg/mL hydrocortisone, 2.5 mM Y-27632 (Focus Biomolecules, Plymouth, PA, USA), and penicillin/streptomycin. DMEM supplemented with 10% FBS and penicillin/streptomycin was used to maintain 293 T cells (RIKEN BioResource Center, Kyoto, Japan). Het-1A cells were purchased from the American Type Culture Collection (ATCC; Manassas, VA, USA) and maintained in airway epithelial cell basal medium from the airway epithelial cell growth medium supplement pack (PromoCell, Heidelberg, Germany) supplemented with 4% FBS and penicillin/streptomycin. IMR-32 cells were purchased from ATCC and maintained in DMEM supplemented with 10% FBS and 0.1 mM non-essential amino acids (Thermo Fisher Scientific, Waltham, MA, USA). HSC-3 cells were purchased from RIKEN (Saitama, Japan) and maintained in Eagle’s minimal essential medium supplemented with 10% FBS and penicillin/streptomycin.

### Cell Sorting

HPCM2 cells were stained with anti-CD271 antibody (ME20.4; BioLegend, San Diego, CA, USA) and sorted according to the CD271 expression (MA900, SONY, Tokyo, Japan). Five percent of low CD271-expression population was collected and subsequently cultured for three days, and total RNA was extracted.

### Establishment of CD271-promoter-deleted mutant cells

We designed guide (g)RNA to delete the RELA-binding site using CRISPRdirect^14^. The gRNAs were inserted into the pSpCas9(BB)-2A-GFP (PX458) vector [a gift from Feng Zhang (Addgene plasmid #48138; Addgene, Watertown, MA, USA)]^15^. The gRNA sequences for deletion of site + 1045 were as follows: 5′-tggggctgcggatctaaggc-3′ and 5′-ggggagtgcccacttcgccg-3′. HPCM2 cells were seeded in two-dimentional culture system, and synchronized with 3 μM aphidicolin (011-09811; Wako, Osaka, Japan) for 24 h prior to targeting^16^. Then both plasmids were transfected into HPCM2 cells using FuGeneHD (Promega, Madison, WI, USA), and for the control, a PX458 vector without gRNA was transfected. Cells were released for 4 h prior to transfection by washing with complete medium, then transfected. At 2-days post-transfection, GFP-positive cells were sorted using a cell sorter (MA900; Sony Biotechnology, Tokyo, Japan) to establish a stable transfectant.

### Cloning of CD271 promoter sequence.

CD271 promoter region was cloned from HPCM2 genome using PCR-based method. Deletion mutants and point mutations of pNL1.1-neo-CD271promotor were generated using PrimeSTAR Mutagenesis Basal Kit (Takara, Osaka, Japan.). Summary of the point mutations are listed in Table 1.

**Table 1:** Sequences of RELA binding sites of CD271 promoter region.

Name	Wild type	Mutant
959 m	tGGACATTTCcag	tGAACATGTccag
1062 m	acttcgccGGGGCGAACCcg	acttcgccGGAGCGAAACcg
1045 m	ggctgggagGGGAGTGCCCac	ggctgggagGGGCGTGCCAAc
1079 m	tcccGGGGTTCCCCcacggc	tcccGGAGTGCCCCcacggc
463 m	gagGGGTCTTTCAagagggggcatgggg	gagGGATCTGTCAagagggggcatgggg
484 m	catGGGGCTCTCCgatgcccaggttcttc	catGGAGCTATCCgatgcccaggttcttc
541 m	cgaaGGGACTTTCCcctcagcatctcggtctct	cgaaGAGACTGTCCcctcagcatctcggtctct
657 m	gcGGGGAGCCCGggacgacg	gcGGAGAGCACgggacgacg

### Prediction of transcription factors

HPCM2 cells were sorted according to CD271 expression using a MA900 (SONY, Tokyo, Japan), and a CD271-low fraction was obtained (day 0). The sorted cells were cultured with RPMI-1640 containing 10% FBS, and cells were obtained after 1, 2, and 3 days in duplicate. The cells were lysed, and total RNA was obtained using an RNeasy micro kit (Qiagen, Hilden, Germany). Microarray analysis (8 × 60 k; Agilent Technologies, Santa Clara, CA, USA) was performed according to manufacturer instructions, and significantly enriched gene sets were determined using Gene Set Enrichment Analysis (GSEA)^17^ by comparing day 0/1 and day 2/3 samples.GSEA was performed by using GSEA software (Broad Institute, https://www.gsea-msigdb.org/gsea/). Enrichment score (ES) reflects the degree to which a gene set is overrepresented at the top or bottom of a ranked list of genes. A positive ES indicates gene set enrichment at the top of the ranked list; a negative ES indicates gene set enrichment at the bottom of the ranked list.

### Prediction of RELA-binding sites

RELA-binding sites in CD271 promoter were predicted using TFBIND^18^. CD271 promoter sequence between 432 and 1138 was analyzed in the website (https://tfbind.hgc.jp/), and RELA binding sites were identified.

### siRNA

Negative control siRNA, RELA siRNA#1 (s535313), and #2 (s11914) were purchased from Thermofisher Science. The siRNA transfections were performed using Lipofectamine RNAiMAX Reagent (Life Technologies, CA, USA) as described previously^19^.

### Plasmid

*RELA, NFKB1, and NFKB2* genes was cloned from HPCM2 genome using a PCR method. A *SP1* gene was obtained from RIKEN DNA Bank (IRAL050J02, Tsukuba, Japan). *SP1* and *NFKB2* genes were inserted into 3xFLAG-CMV10 vector, and *NFKB1* and *RELA* were 3xFLAG-CMV14, respectively (Sigma Aldrich, St. Louis, MO, USA).

### 3-(4,5-Dimethylthiazol-2-yl)-2,5-diphenyltetrazolium bromide (MTT) assay

MTT assay was performed as described previously^19^. In brief, MTT (5 mg/mL, 1:10) was added to the medium and after 2 h, equal volume of 10% SDS in 0.01 M HCl was added and further incubate for overnight, and the absorbance value of 550 nm was measured.

### Luciferase assay

The *CD271* promoter region was amplified from the genome of HPCM2 cells using polymerase chain reaction and cloned into the pNL1.1-Nluc-Neo vector (Promega). 293 T cells were transfected with the plasmids using FugeneHD (Promega) according to manufacturer instructions, and 2 days after transfection, cells were lysed with Nanoglo (Promega), and luminescence was measured using a Synergy H1 system (Agilent Technologies). The pGL4.10[luc2] vector (Promega) was also transfected along with pNL1.1-Nluc-Neo for normalization.

### Sphere-formation assay

1 × 10^3 cells were cultured with DMEM/F12 supplemented with B27 (1:50; Thermo Fisher Scientific), epidermal growth factor (20 ng/mL; Peprotech, Cranbury, NJ, USA), and fibroblast growth factor-2 (20 ng/mL; Peprotech) on a 96-well Nunclon Sphera plate (Thermo Fisher Scientific). After 7 days, MTT assay was performed as described previously with minor modifications^20^. A 1/10 volume of MTT assay reagent (5 mg/mL; Fujifilm Wako Pure Chemical Corp.) was added to each well and incubated in a humidified 5% CO_2_ incubator. After 2 h, 10% SDS in 0.01 M HCl was added to each well and incubated overnight at 37 °C. The absorbance at 575 nm and 650 nm (background measurement) was determined using a VersaMax ELISA Microplate Reader (Molecular Devices, Sunnyvale, CA, USA).

### Flow cytometry

Flow cytometry was performed as previously described^8^. Fluorescence data were collected using a FACSCanto II system (BD Biosciences), a SA3800 cell analyzer (Sony Biotechnology, Tokyo, Japan), or a MA900 cell sorter (Sony Biotechnology) according to staining using an anti-CD271 antibody (ME20.4; BioLegend, San Diego, CA, USA). Data were analyzed using FlowJo software (v10, FlowJo LLC, Ashland, OR, USA) and the CytoExploreR package in R software^21^.

### Western blot

Western blot was performed as previously described^8^ using the following antibodies: anti-CD271 (1:1000; D4B3; Cell Signaling Technology, Danvers, MA, USA), anti-RELA (1:1000; D14E12; Cell Signaling Technology), horseradish peroxidase (HRP)-conjugated anti-β-actin (1:1000: Medical and Biological Laboratories Co., Ltd., Nagoya, Japan), anti-α-tubulin (1:1000; Medical and Biological Laboratories Co., Ltd.), HRP-conjugated anti-mouse IgG (1:1000; Cell Signaling Technology), and HRP-conjugated anti-rabbit IgG (1:1000; Cell Signaling Technology). The samples were separated by sodium dodecyl sulfate polyacrylamide gel electrophoresis (Bio-Rad, Hercules, CA, USA) and transferred onto polyvinylidene fluoride membranes (Bio-Rad).

### Immunohistochemistry

Tumor specimens were obtained from 38 consecutive cases from 2013 to 2017 of hypopharyngeal cancer at Miyagi Cancer Center (Natori, Japan), in which patients underwent biopsy before treatment. All of the cases were pathologically diagnosed as squamous cell carcinoma. Immunostaining of formalin-fixed paraffin-embedded tissue was performed as previously described^8^ using the following antibodies: anti-CD271 (1:2500; C40-1457; BD Biosciences), anti-phosphorylated (p)-RELA (1:1500; ab86299; Abcam, Cambridge, UK), and EnVision + Dual Link System-HRP (anti-mouse and anti-rabbit; Dako, Glostrup, Denmark). Heat-induced epitope retrieval was performed by microwaving the sections in target retrieval solution for CD271 staining (pH 9.0; Dako) or Immunosaver (Fujifilm Wako Pure Chemical Corp., Osaka, Japan) for p-RELA staining.

### Analysis of the cancer genome atlas (TCGA) data

An RNA-seq dataset (normalized using fragments per kilobase of transcript per million mapped reads) for HNSC was downloaded from TCGA (https://portal.gdc.cancer.gov/). After excluding normal tissue samples, a total of 500 cases were enrolled. The cases were divided into CD271-high and -low groups by median expression value, and gene set enrichment analysis (GSEA) was performed^17^.

### Statistical analysis

Significant differences between experimental groups were determined by Student’s *t* test using GraphPad Prism software (v9.0; GraphPad Software, San Diego, CA, USA) or R software (v4.1.0)^22^, with a p < 0.05 considered significant.

## Results

### Identification of transcription factors targeting the CD271 promoter

**Figure 1:**
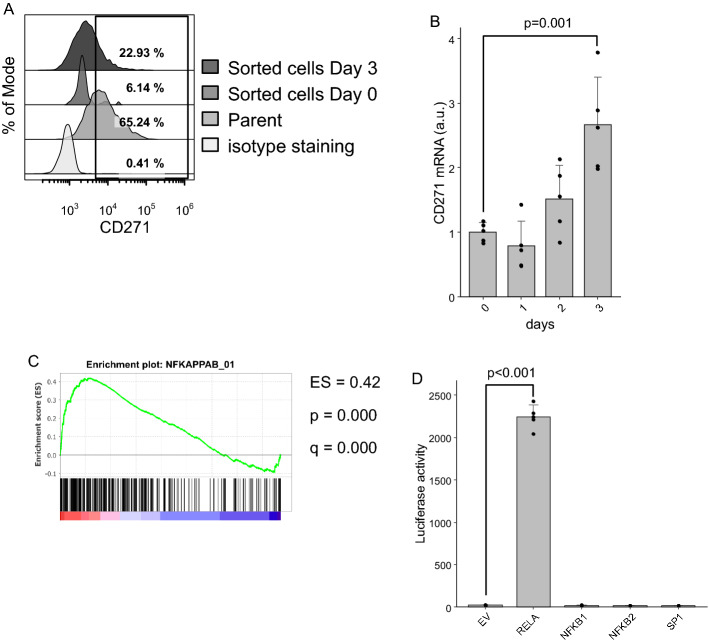
Screening transcription factors potentially involved in *CD271* expression. (**A**) Representative data of *CD271* expression in HPCM2 cells. HPCM2 cells were sorted into a CD271-low fraction and cultured for 3 days, followed by determination of CD271 expression using flow cytometry. (**B**) Real-time PCR of CD271 mRNA. The sorted cells (day-0) and subsequently cultured cells (day-1, 2, and 3) were harvested and total RNA was purified. (**C**) Gene Set Enrichment Analysis. Comprehensive gene expressions were obtained using microarray and compared day-2, 3 versus 0, 1. Target genes of NFKB and RELA were upregulated in day-2, 3 cells. ES, enrichment score. (**D**) Luciferase assay. 293 T cells were transfected with vectors harboring the indicated genes and the *CD271* promoter region containing a luciferase reporter. Luciferase activity was measured 2-days post-transfection.

To identify transcription factors targeting the *CD271* promoter, we compared sorted CD271-low HPCM2 cells with those showing proliferation after sorting according to the cell surface CD271 expression (Fig. 1A). Sorting of HPCM2 according to low CD271 expression resulted in recovery of CD271 protein (Fig. 1A) and mRNA (Fig. 1B) expression after 3 days (Fig. 1B), which is consistent with our previous data^7^, enabling comparison of transcriptomes between CD271-low and CD271-recovered fractions in order to identify changes in *CD271*-specific transcription factor activity. We obtained comprehensive gene expression and compared between day-0, 1 and 2, 3 samples using Gene Set Enrichment Analysis (GSEA)^17^, and found that the NFKAPPAB_01 gene set, which contains genes having the motif of RELA and NFKB transcription factor binding sites, was significantly enriched in day 2 and 3 samples (Fig. 1C). To validate this result, we cloned the CD271 promoter region and examined promoter activity using 293 T cells overexpressing each transcription factor. Although we observed no enhanced luciferase activity in NFκB1 and NFκB2-overexpressing cells, RELA-overexpressing cells showed prominent luciferase activity (Fig. 1D). Additionally, we confirmed that Sp1, previously reported as a transcription factor of CD271 in neuroblastoma cells^8^, did not initiate luciferase activity.

### RELA induces CD271 expression in squamous cell carcinoma cells

**Figure 2:**
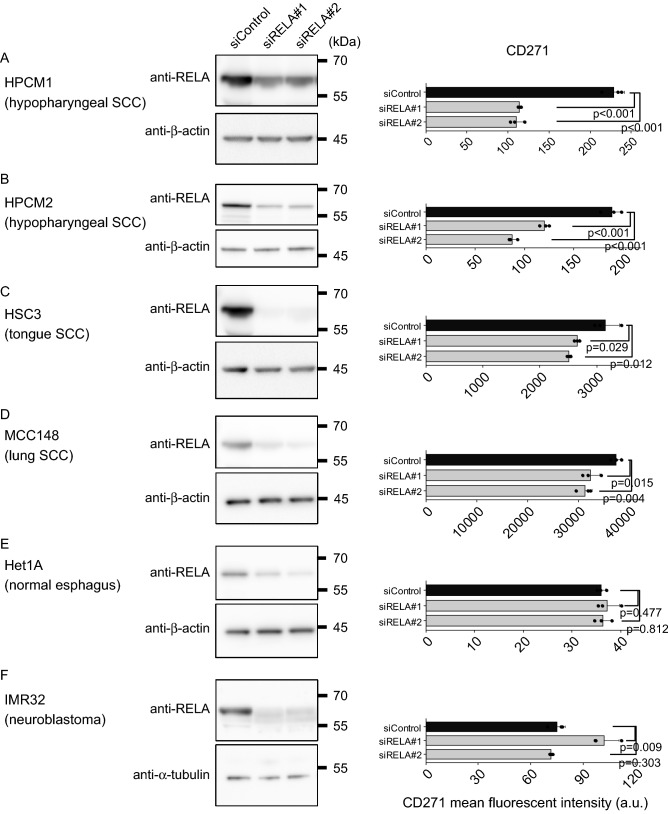
RELA is required for *CD271* expression in squamous cell carcinoma cell lines. (**A**)–(**F**) Cells were transfected with siRNA targeting *RELA*, followed by western blot of RELA and flow cytometric analyses of CD271 expression. SCC, squamous cell carcinoma.

We then validated RELA-specific promotion of *CD271* expression in HNSC cells. In HPC cell lines (HPCM1 and HPCM2; both squamous cell carcinoma), we observed significant decreases in *CD271* expression following transfection with small-interfering (si)RNA targeting *RELA* (Fig. 2A,B, and Supplemental Fig. 1), with similar results observed in HSC3 cells (tongue squamous cell carcinoma). We previously reported that CD271 plays a critical role in lung squamous cell carcinoma ^5^; therefore, we performed this assay using MCC148c cells (lung squamous cell carcinoma) and observed the same results. However, in normal esophageal epithelium (Het-1A) and a neuroblastoma cell line (IMR-32), RELA knockdown did not affect *CD271* expression, despite confirmation of decreased RELA levels (Fig. 2 and Supplemental Fig. 1). These data indicated that RELA-dependent *CD271* expression might be specific to squamous cell carcinoma.

**Figure 3:**
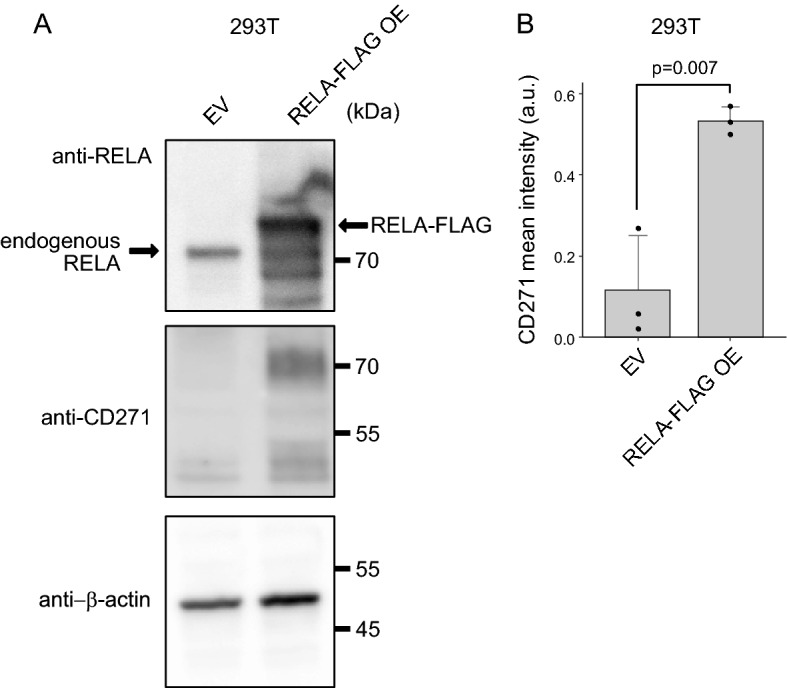
*RELA* overexpression promotes CD271 expression. (**A**) 293 T cells were transfected with a p3xFLAG-CMV14-RELA, and levels of the indicated proteins were determined by western blot using indicated antibodies. (**B**) CD271 expression following p3xFLAG-CMV14-RELA overexpression according to flow cytometry. OE, overexpression.

We then investigated RELA functions using RELA-overexpressing 293 T cells based on their low expression of CD271 and ease of transfection using the p3xFLAG-CMV14-RELA vector. Following transfection, western blot and flow cytometric analyses confirmed a significantly higher CD271-positive population in RELA-FLAG-overexpressing cells relative to control cells in both experiments (Fig. 3A and B).

### Identification of RELA-binding sites in the CD271 promoter region

**Figure 4:**
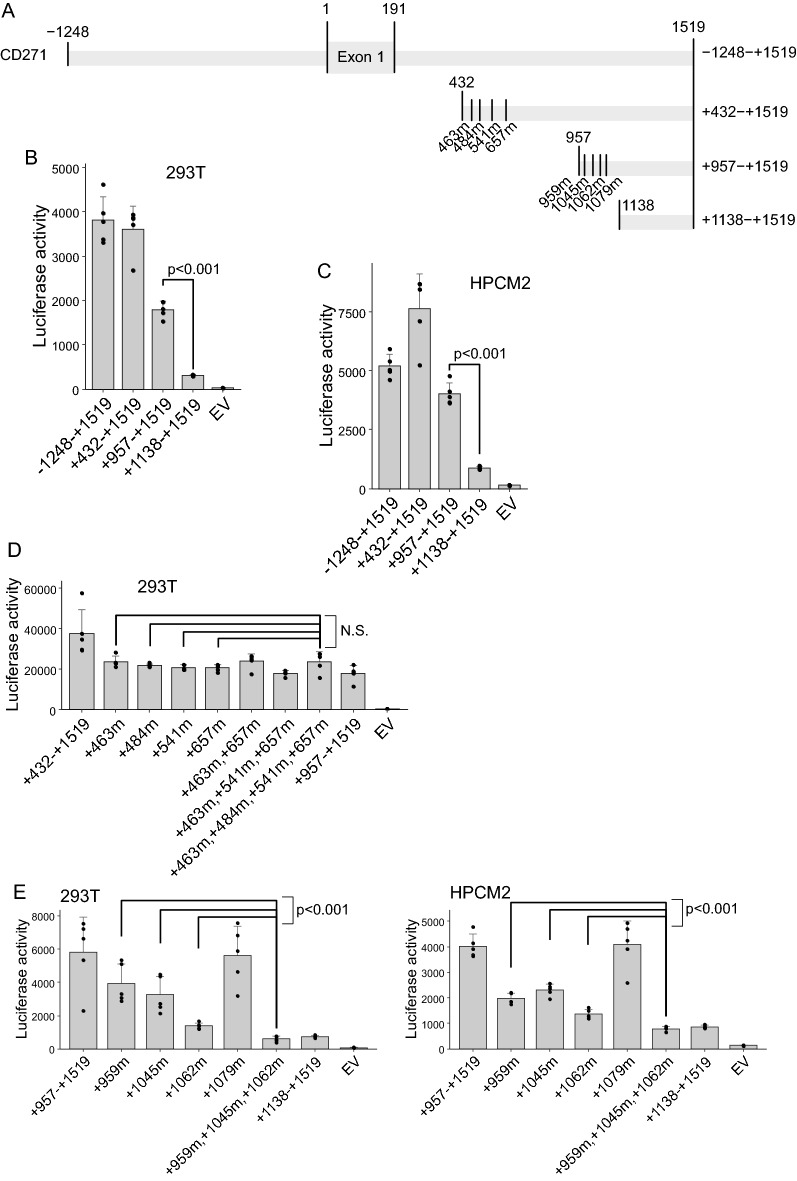
Identification of the RELA-binding site(s) for *CD271* expression. (**A**) Schematic of the *CD271* promoter region and its deletion mutants. (**B**)–(**E**) Luciferase assay. 293 T or HPCM2 cells were transfected with a vector containing the *CD271* promoter region fused along with a luciferase reporter and a *RELA*-expression vector. Luciferase activity was measured two-days post-transfection.

To investigate the specific region in the *CD271* promoter responsible RELA-induced transcription, we introduced mutations into the *CD271* promoter region and performed luciferase assays (Fig. 4A). The results identified decreased luciferase activity in cells harboring regions + 957 to + 1519 and + 1138 to + 1519 (Fig. 4B), with similar results observed in HPCM2 cells (Fig. 4C). A search for RELA-binding sites from site + 432 to + 1138 revealed seven potential binding sites; therefore, we created clones harboring mutations in these specific regions and the luciferase assays. Although luciferase activity was modestly altered in clones harboring mutations between + 432 and + 957, no difference was observed between single mutations and multiple mutations (Fig. 4D). We observed decreased luciferase activity for deletions in the region + 957 to + 1519, with significant decreases in activity observed with mutation at sites + 959, + 1045, + 1062, respectively, and more pronounced decrease of all three sites in combination in 293 T and HPCM2 cells (Fig. 4E).

### Deletion of RELA-binding site decrease sphere forming capacity

**Figure 5:**
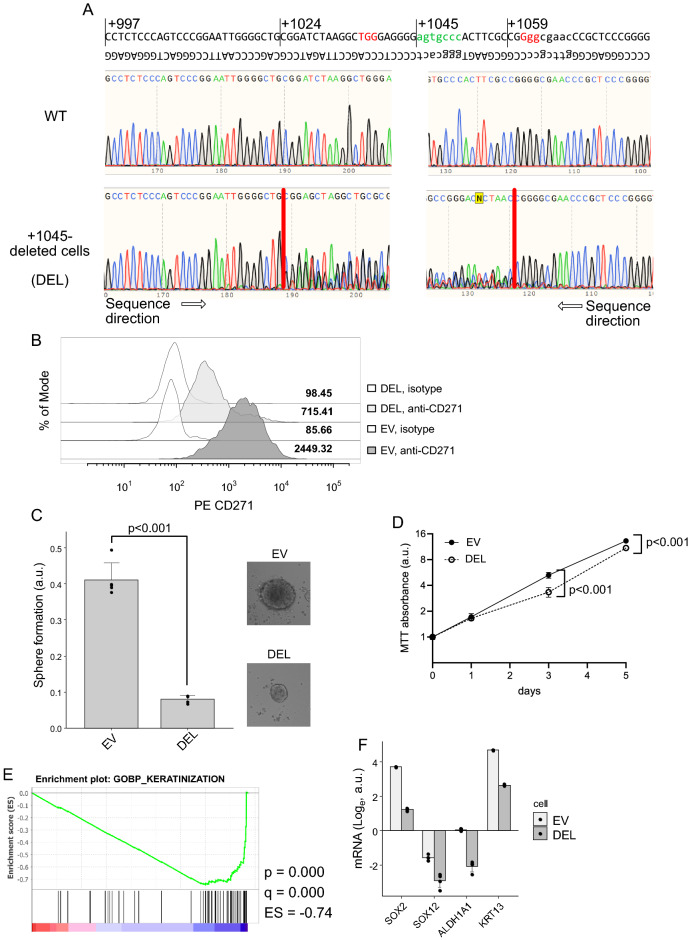
Deletion of the RELA-binding site reduces sphere-formation activity in HPCM2 cells. (**A**) Sequence chromatograms of cells harboring deletion of site + 1045 (DEL). Red characters indicate protospacer adjacent motif sequences, and green characters indicate the + 1045 RELA-binding site. (**B**) CD271 expression according to flow cytometry. The numbers in the histogram indicate the mean fluorescence intensity of phycoerythrin-conjugated anti-CD271. (**C**) Sphere-formation activity of RELA binding site-knock out HPCM2 cells according to MTT assay. DEL, cells harboring deletion of the RELA-binding site (+ 1045); EV, empty vector (control cells). Right panels show the representative images of the spheroid cells. (**D**) Cell proliferation assay. EV and DEL HPCM2 cells were seeded (1000 cells/well) and cultured and cell numbers were determined by MTT assay. (**E**) GSEA analysis. The exression microarray data of EV versus DEL were compared using GSEA software. A gene set of keratinization was downregulated in EV cells.

To investigate whether these RELA-binding sites play critical roles in CD271 function and cancer malignancy (especially cancer stem cell-related phenotypes), we performed a sphere-forming assay using cells harboring mutations of RELA-binding sites in the *CD271* promoter. Although we attempted to establish cells harboring each RELA-binding-site mutation using the CRISPR/Cas9 system, mutation at site + 959 and + 1062 inhibited cell proliferation (data not shown). We successfully established cells harboring deletion of site + 1045 (Fig. 5A), and we subsequently confirmed decreases in CD271 expression in these cells (Fig. 5B). Additionally, sphere-formation assays showed a significant decrease in the number of spheroids formed by the mutant cells relative to controls (Fig. 5C). Moreover, assessment of the proliferative capacity of the mutant cells in two-dimensional culture indicated slightly decreased proliferation by the mutant cells relative to that observed in controls (Fig. 5D). Furthermore, gene-expression analysis revealed enrichment of a gene set named KERATINIZATION, generally known as a differentiation status^23^, in the mutant cells (Fig. 5E). Furthermore, SOX2^24^, SOX12^25^, ALDH1A1^26^, and KRT13^27^ genes were downregulated in CD271 promoter knock-out cells (Fig. 5F), suggesting loss of stemness by depletion of CD271.

### Relationships between CD271 and RELA in clinical specimens

**Figure 6:**
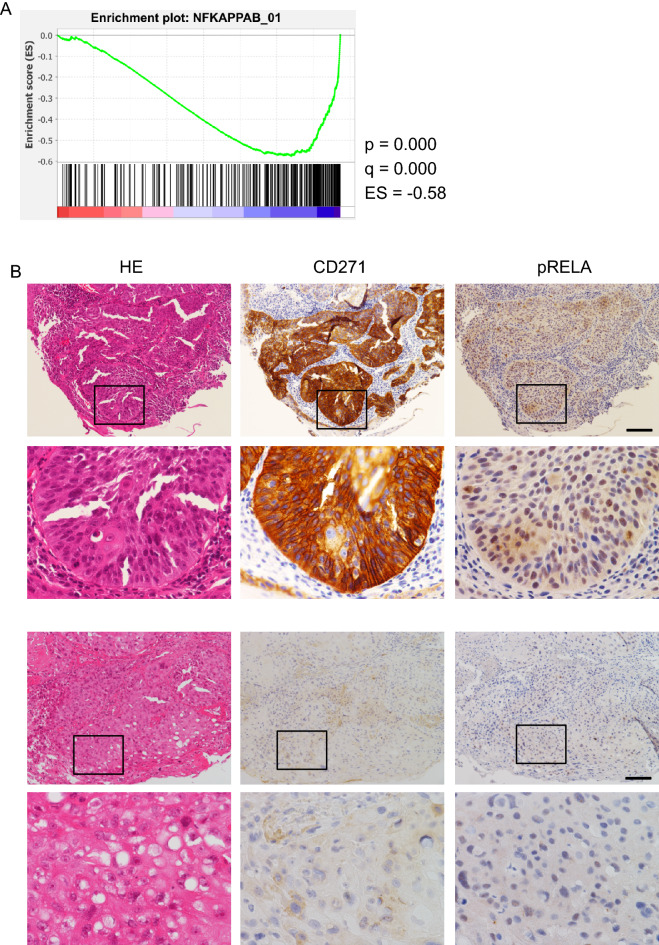
Correlation of RELA and CD271 levels in clinical specimens. (**A**) Enrichment plot calculated using GSEA software. HNSC data from TCGA was divided into CD271-high and -low samples, and RELA-binding sites were identified. ES, enrichment score. (**B**) Surgical resection specimens were stained with anti-CD271 or anti-p-RELA. Upper panels show a sample where both CD271 and p-RELA were upregulated, and the lower panel shows a sample where neither was expressed. Bar, 100 mm.

To elucidate a relationship between RELA and CD271 in clinical specimens, we searched TCGA and performed RNA-seq analysis. Because RELA phosphorylation is required for its transcriptional activity^28^, we investigated correlations between *CD271* transcript levels and those of other RELA-targeted genes. The results identified significant enrichment of a gene set (NFKAPPAB_01) containing genes harboring NF-κB- and RELA-binding sites, among CD271-high cases (Fig. 6A). Subsequent immunohistochemistry analysis of CD271 and RELA expression in clinical specimens revealed that CD271 and p-RELA staining were significantly correlated and pp65-high cases tended to be CD271-high (Fig. 6B and Table 2).

**Table 2:** Characteristics of patients with gastric cancer.

CD271	− (*N* = 17)	+ (*N* = 20)	*p*-value^1^
Age, Median (IQR)	69 (68–73)	69 (63–71)	0.17
Gender (%)			> 0.99
Female	1 (5.9)	2 (10)	
Male	16 (94)	18 (90)	
pp65 (%)			0.014
−	5 (29)	0 (0)	
+	12 (71)	20 (100)	
cSTAGE (%)			0.38
1	2 (12)	5 (25)	
2	3 (18)	4 (20)	
3	0 (0)	2 (10)	
4	12 (71)	9 (45)	
cT (%)			0.84
T1	3 (18)	6 (30)	
T2	6 (35)	7 (35)	
T3	4 (24)	4 (20)	
T4	4 (24)	3 (15)	
cN (%)			0.61
N0	7 (41)	11 (55)	
N1	0 (0)	1 (5.0)	
N2	8 (47)	7 (35)	
N3	2 (12)	1 (5.0)	
cM (%)			0.2
M0	15 (88)	20 (100)	
M1	2 (12)	0 (0)	

^1^Wilcoxon rank sum exact test; Fisher's exact test.

## Discussion

In this study, we identified RELA as a transcription factor of *CD271*, as well as the target region for RELA binding in the CD271 promoter. Additionally, we confirmed that deletion of this region reduced spheroid formation, a model of cancer stem cell enrichment^29^, although the proliferative capacity of the cells in 2D culture was only slightly decreased, suggesting that CD271 affect stemness rather than cell proliferation. Moreover, GSEA results identified enrichment of a gene set related to KERATINIZATION, which is related to differentiation in squamous cell carcinoma. We previously reported that CD271 plays critical roles in cancer stem cells^7^. The present data suggest that *CD271* expression induced by RELA binding at the + 1045 site in the CD271 promoter is involved in cancer stemness, as well as cell proliferation. Furthermore, we confirmed these findings using several squamous cell carcinoma cell lines. In addition, we verified that RELA was not involved in *CD271* expression in neuroblastoma or normal epithelial cell lines, which differed from a previous study suggesting that Sp1 is important for *CD271* transcription in neuroblastoma cells^9^. In the present study, we showed that RELA-specific transcription of *CD271* is cell-type-dependent.

Although harboring deletion of site + 1045 resulted in reduced sphere-formation activity, we were unable to establish cultures of cells harboring deletion of site + 959 and + 1062. We previously showed that the proliferative capacity of CD271 knockdown cells is dramatically reduced in HPCM2 cells^8^. Therefore, it is possible that RELA binding at sites + 959 and + 1062 are more critical than + 1045 for cell proliferation.

NF-κB is a member of a family of transcription factors involved in regulating a wide variety of biological responses^30^. In HNSC, the NF-κB pathway is often activated along with cancer development and progression^31^. In HPC cell lines, previous report demonstrated increased RELA phosphorylation following stimulation with bile acid, which is a risk factor for upper aerodigestive tract malignancies^32^. Moreover, in the present study, we identified RELA as a transcription factor for *CD271* and that deletion of RELA-binding sites reduced spheroid-forming activity. These data are compatible with previous studies and suggest that inhibiting RELA binding to the *CD271* promoter could represent a promising therapeutic target.

## Supplementary Information

Supplementary Figure S1.

Supplementary Figures.

## Data Availability

The microarray datasets generated and analyzed during the current study are available in Gene Expression Omnibus (https://www.ncbi.nlm.nih.gov/geo/, GSE212399).
