# Supplementary material for: RELA is required for CD271 expression and stem-like characteristics in hypopharyngeal cancer
Source: Sci Rep. 2022 Oct 22;12:17751. doi: 10.1038/s41598-022-22736-6 (PMC9588052; doi:10.1038/s41598-022-22736-6)

Supplemental Figure 1

Cells were transfected with siRNA targeting RELA, and on day 1 (HPCM2, MCC148) or day2 (HPCM1, HSC3, Het1A, and IMR32) cells were harvested and CD271 mRNA was measured by real-time PCR.

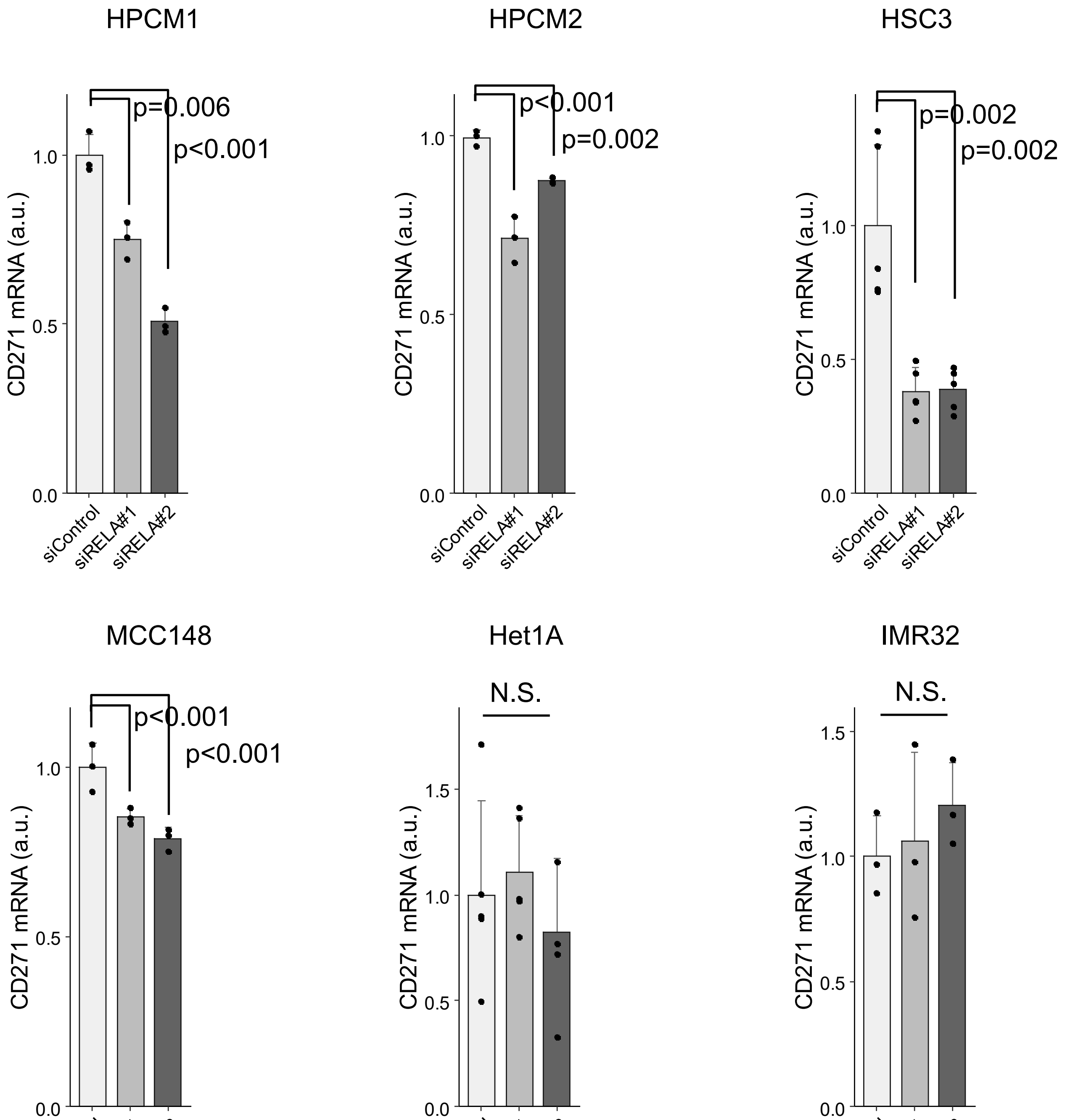

Supplement: Supplementary file 1 — Supplementary Figure S1. [file 41598_2022_22736_MOESM1_ESM.pdf]
