# Supplementary material for: RELA is required for CD271 expression and stem-like characteristics in hypopharyngeal cancer
Source: Sci Rep. 2022 Oct 22;12:17751. doi: 10.1038/s41598-022-22736-6 (PMC9588052; doi:10.1038/s41598-022-22736-6)

Supplemental Figure 2 Full-size images of western blotting

Fig. 2

A

HPCM1
(hypopharyngeal SCC)

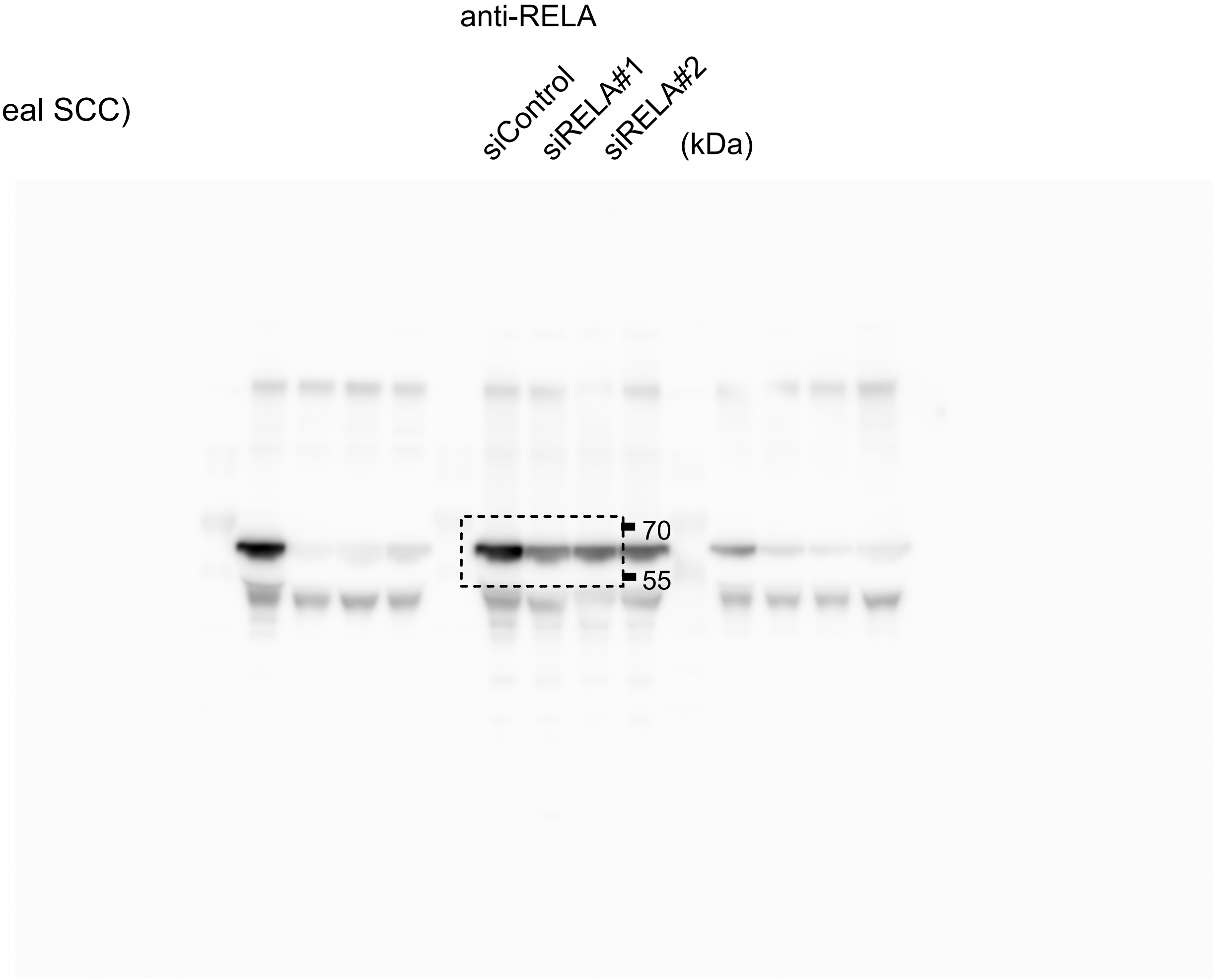

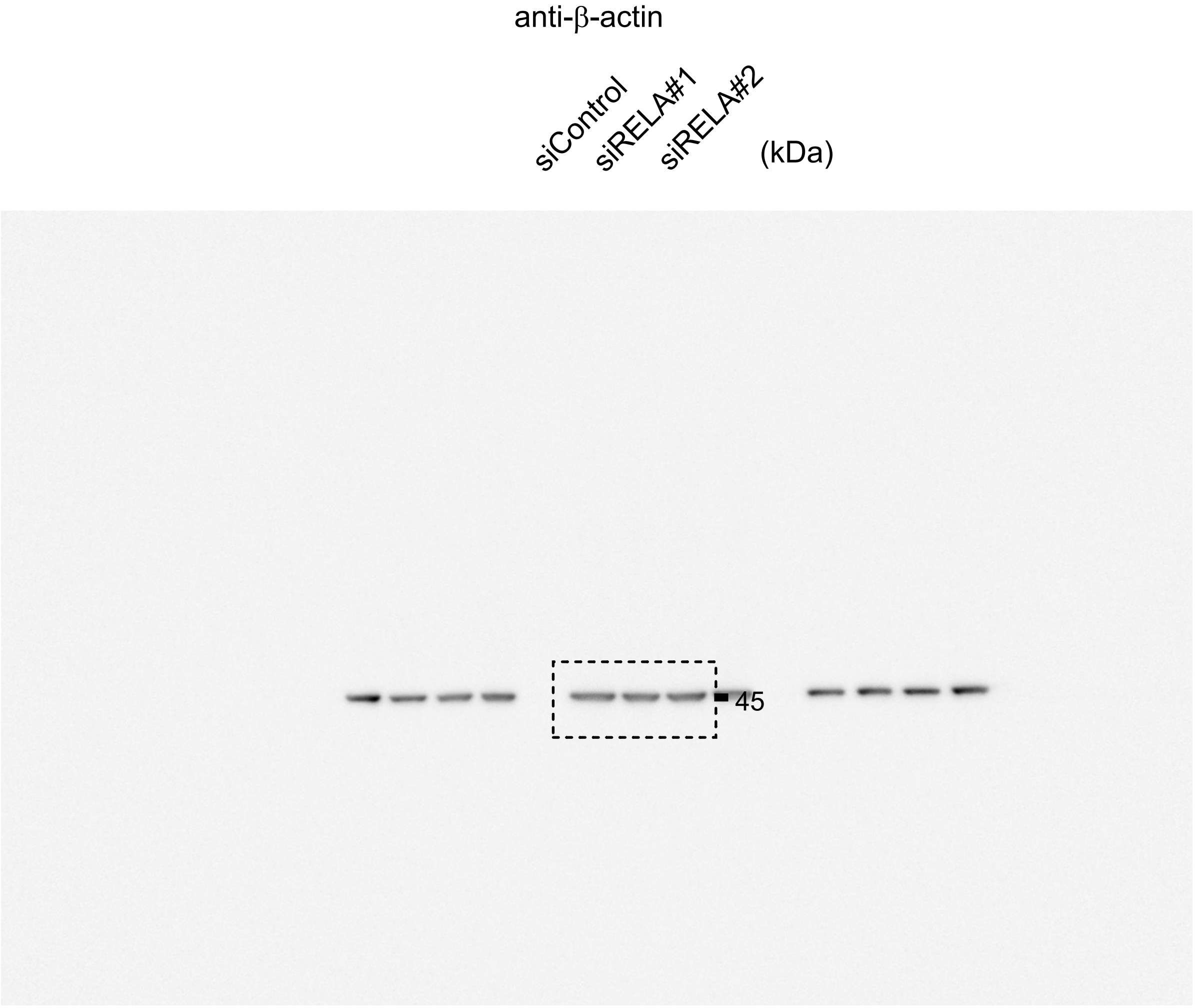

Supplemental Figure 2 Full-size images of western blotting

Fig. 2

A

HPCM1
(hypopharyngeal SCC)

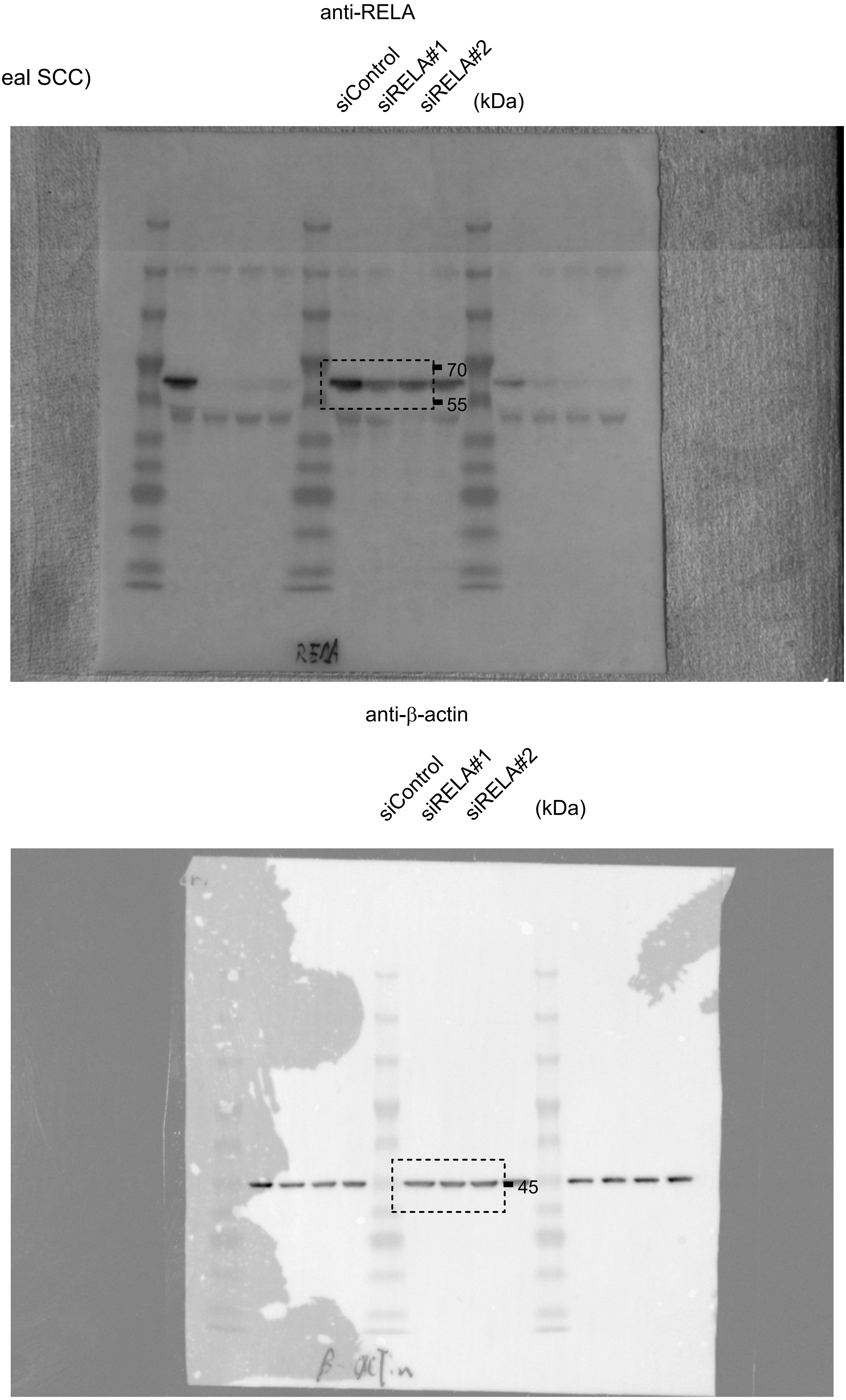

Supplemental Figure 2 Full-size images of western blotting

Fig. 2

B

HPCM2
(hypopharyngeal SCC)

anti-RELA

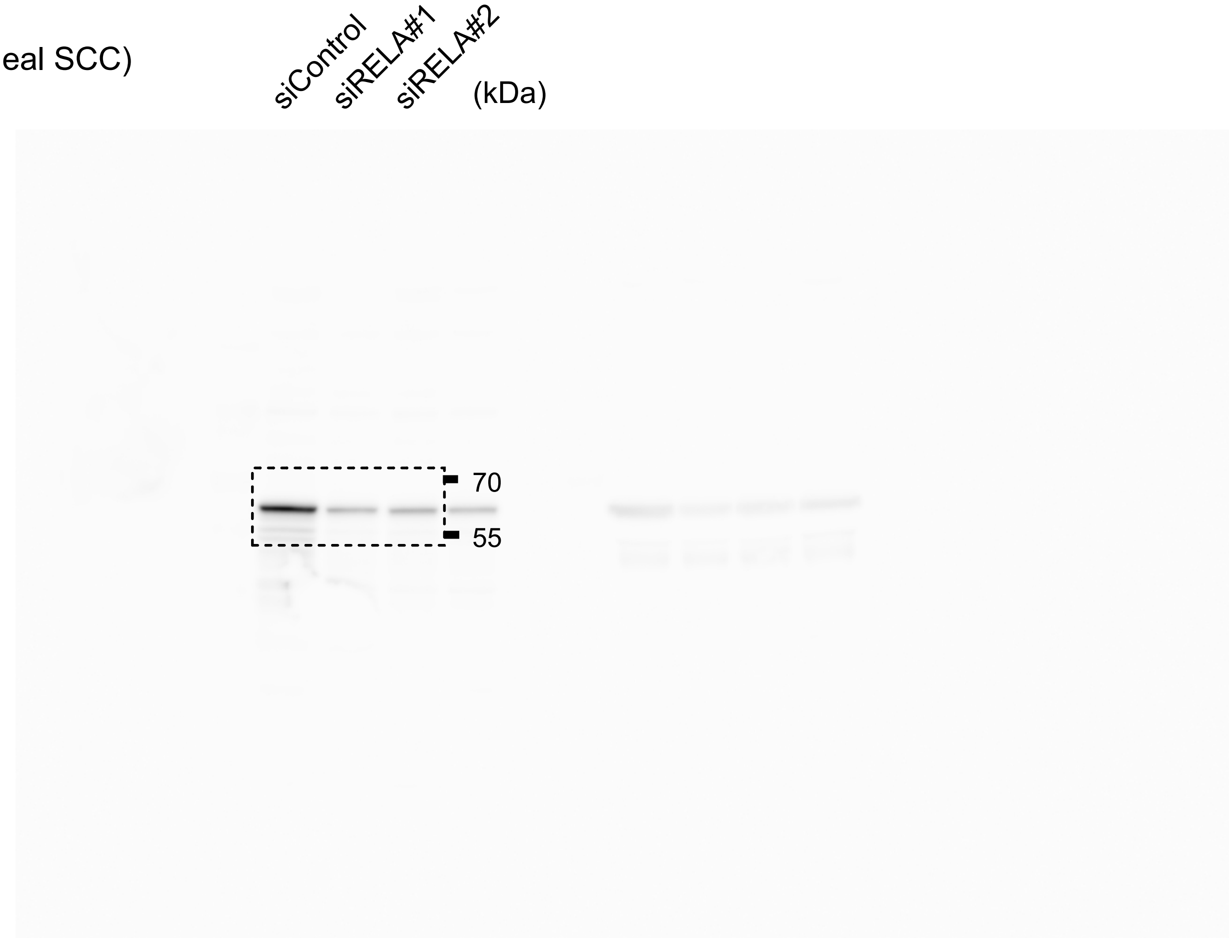

anti-β-actin

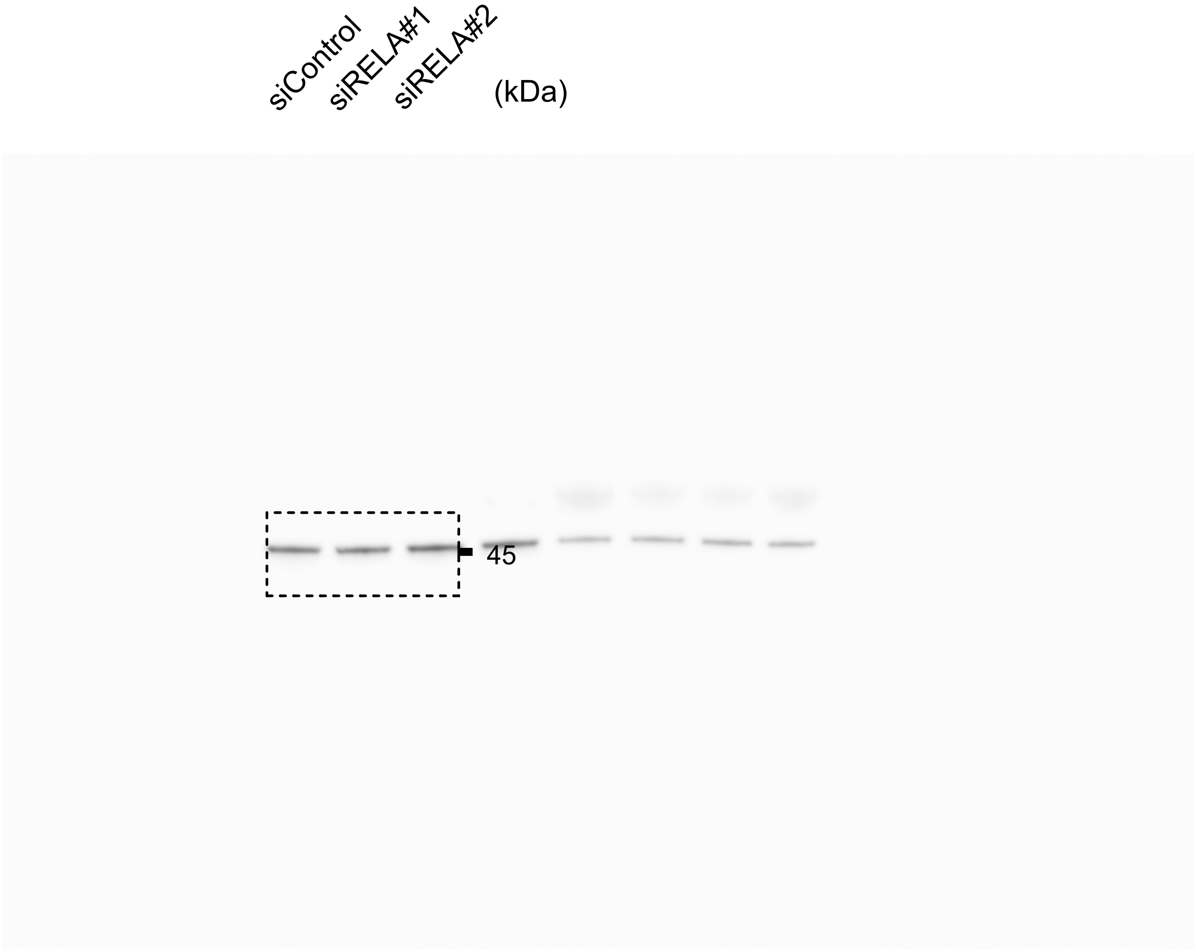

Supplemental Figure 2 Full-size images of western blotting

Fig. 2

B

HPCM2
(hypopharyngeal SCC)

anti-RELA

siControl siRELA#1 siRELA#2 (kDa)

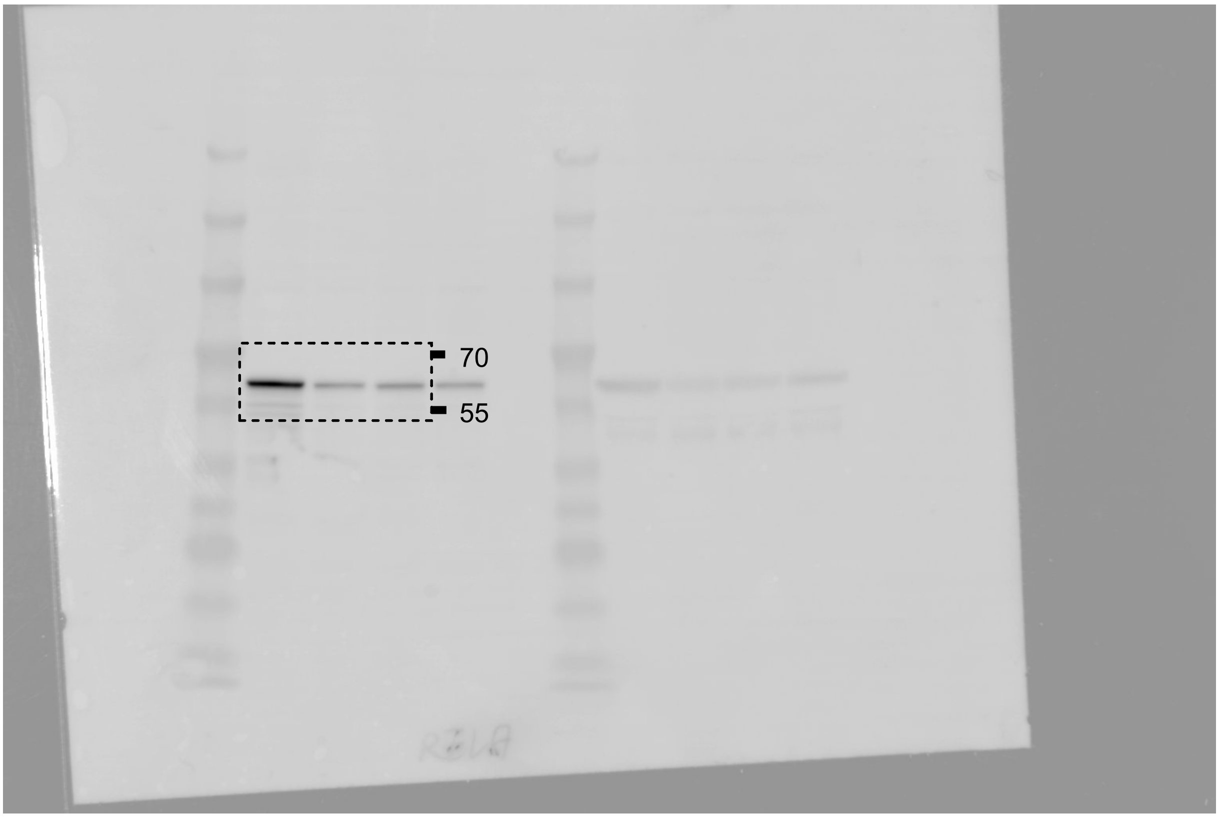

anti-β-actin

siControl siRELA#1 siRELA#2 (kDa)

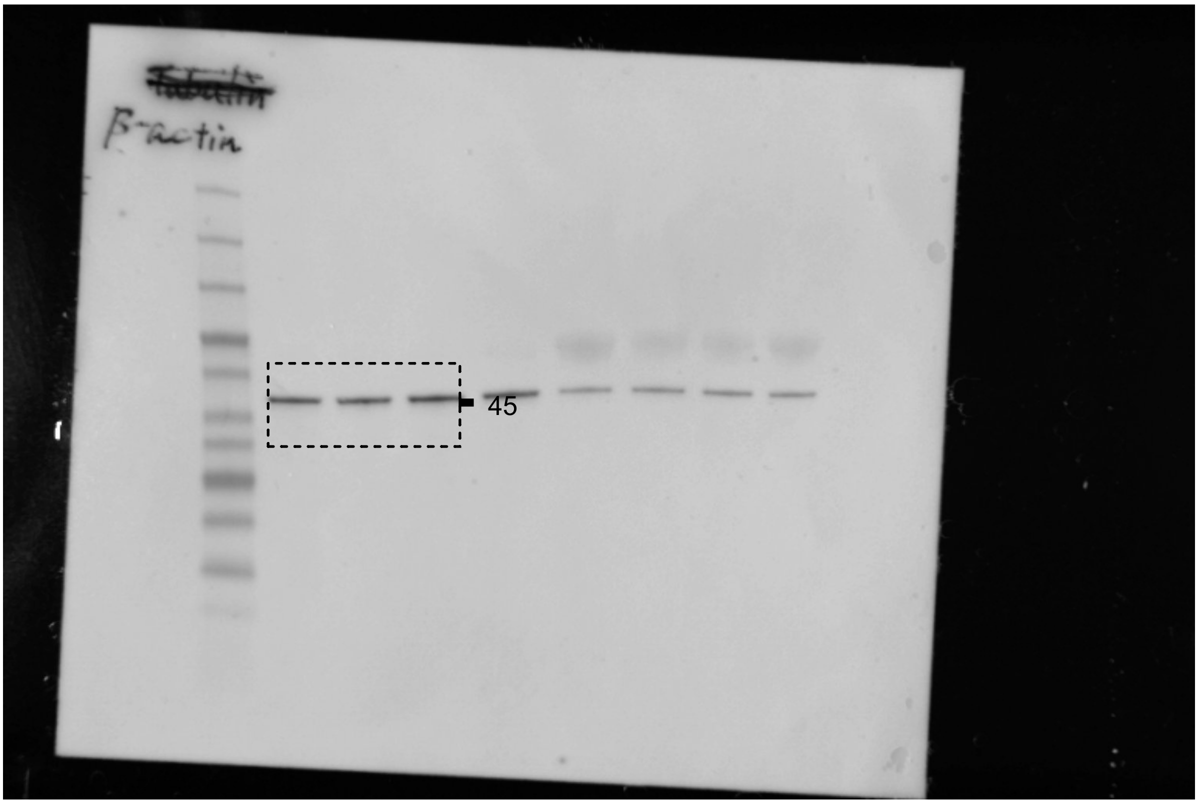

Supplemental Figure 2 Full-size images of western blotting

Fig. 2

C

HSC3
(tongue SCC)

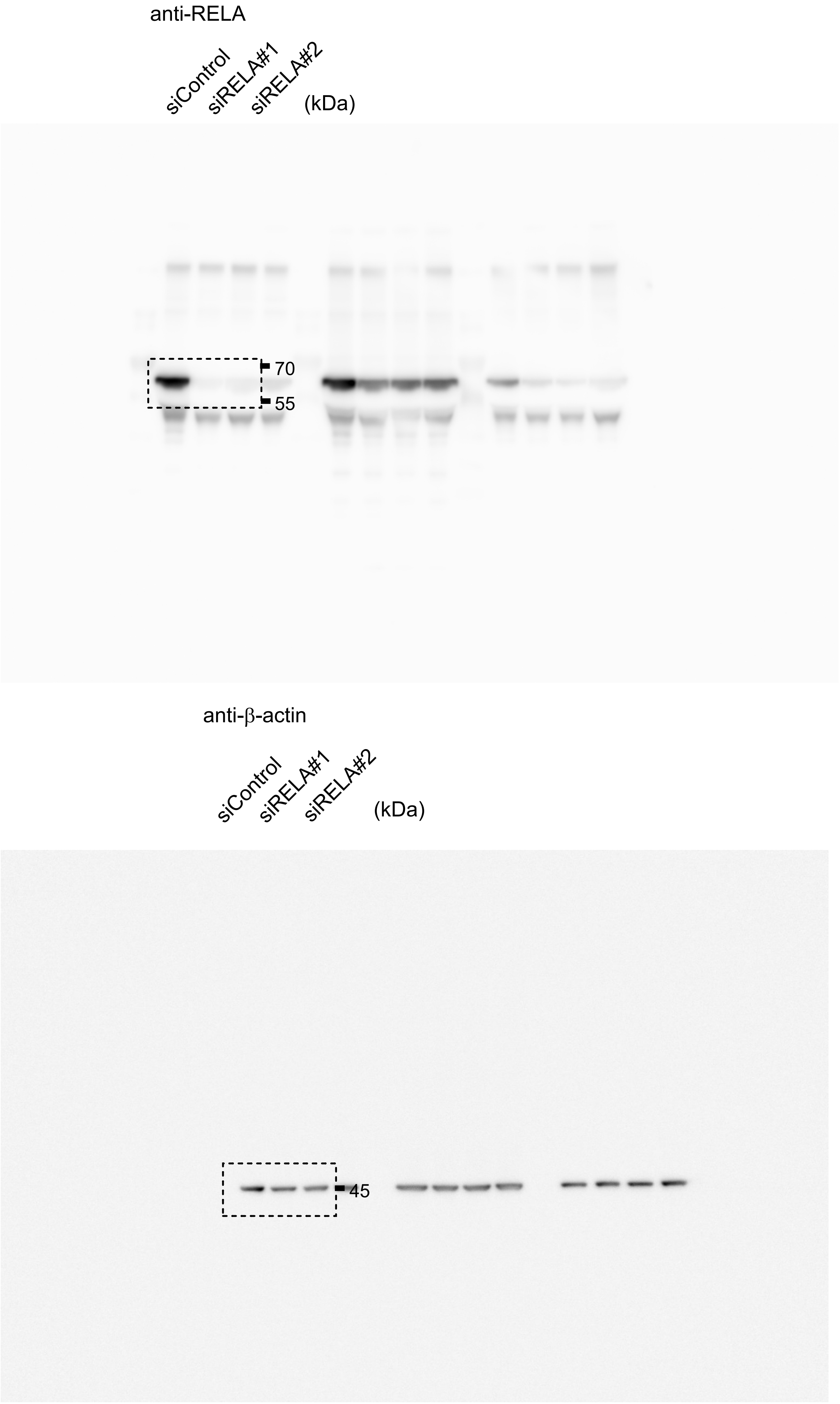

Supplemental Figure 2 Full-size images of western blotting

Fig. 2

C

HSC3
(tongue SCC)

anti-RELA

siControl siRELA#1 siRELA#2 (kDa)

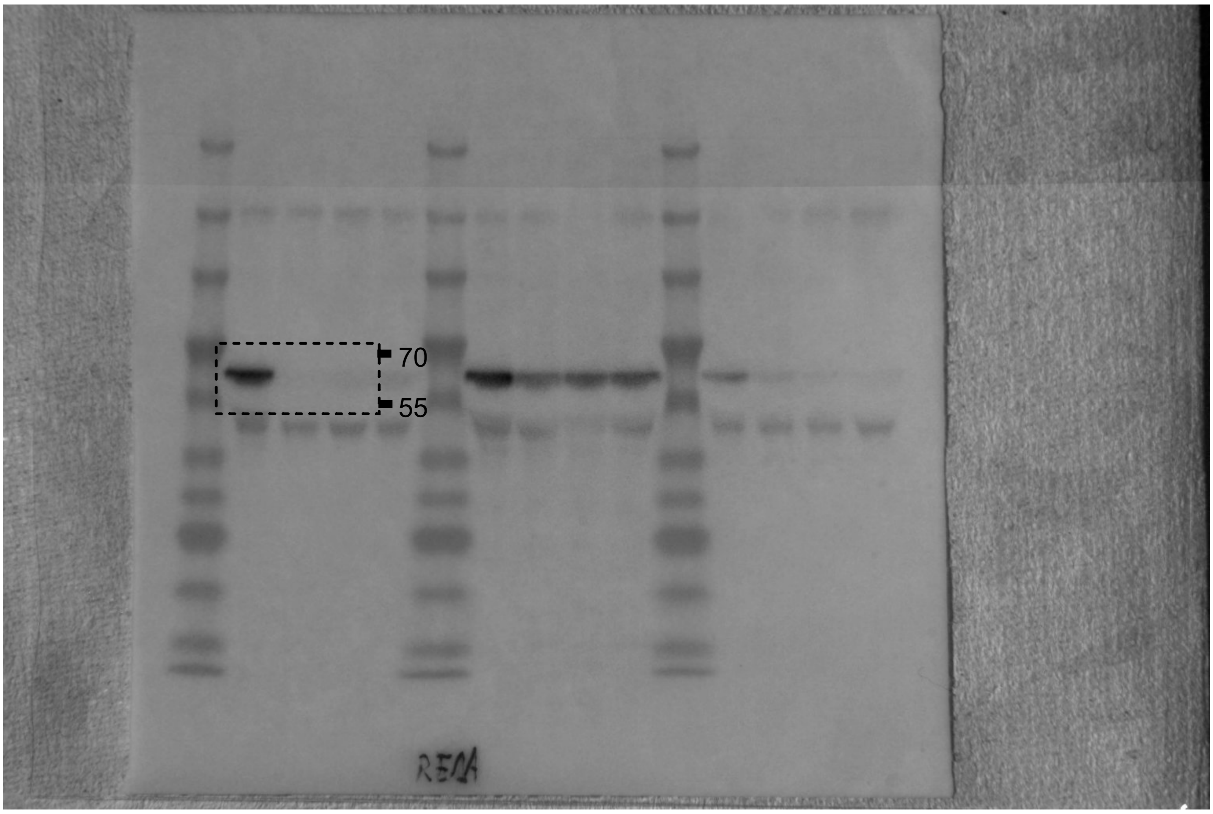

anti-β-actin

siControl siRELA#1 siRELA#2 (kDa)

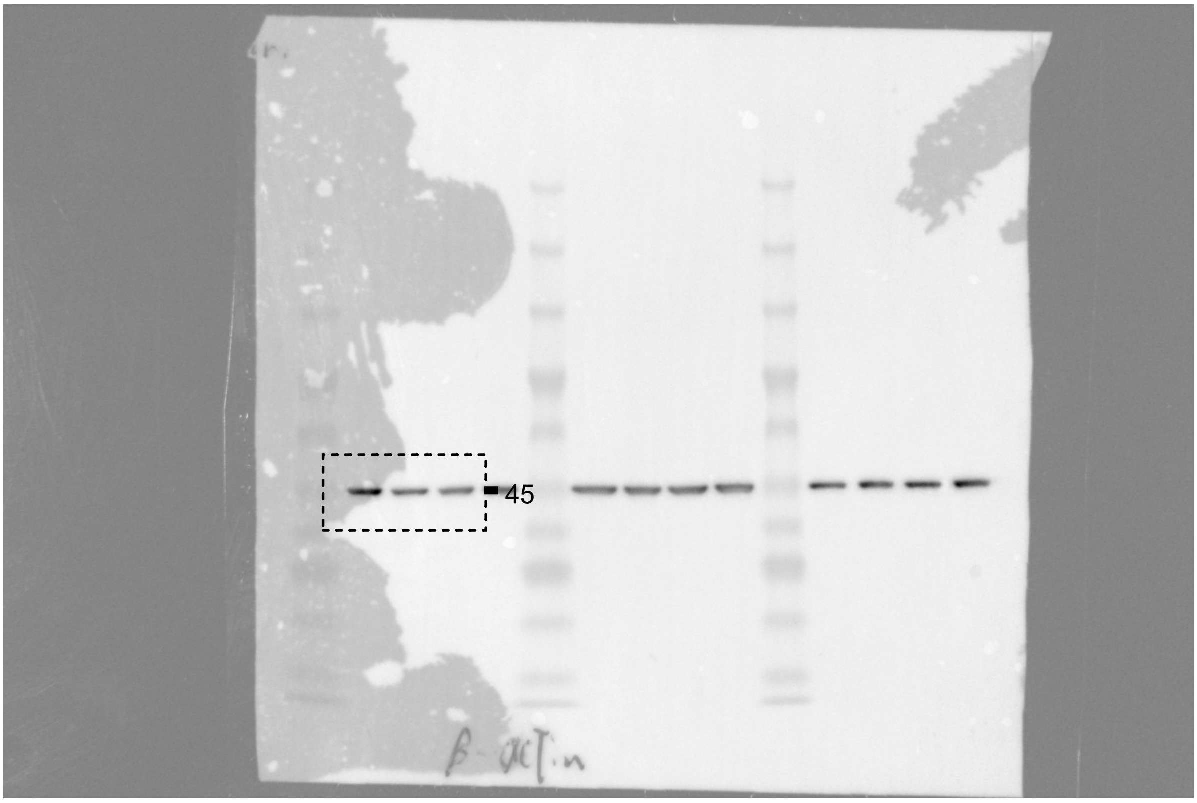

Supplemental Figure 2 Full-size images of western blotting

Fig. 2

D

MCC148
(lung SCC)

anti-RELA

siControl siRELA#1 siRELA#2 (kDa)

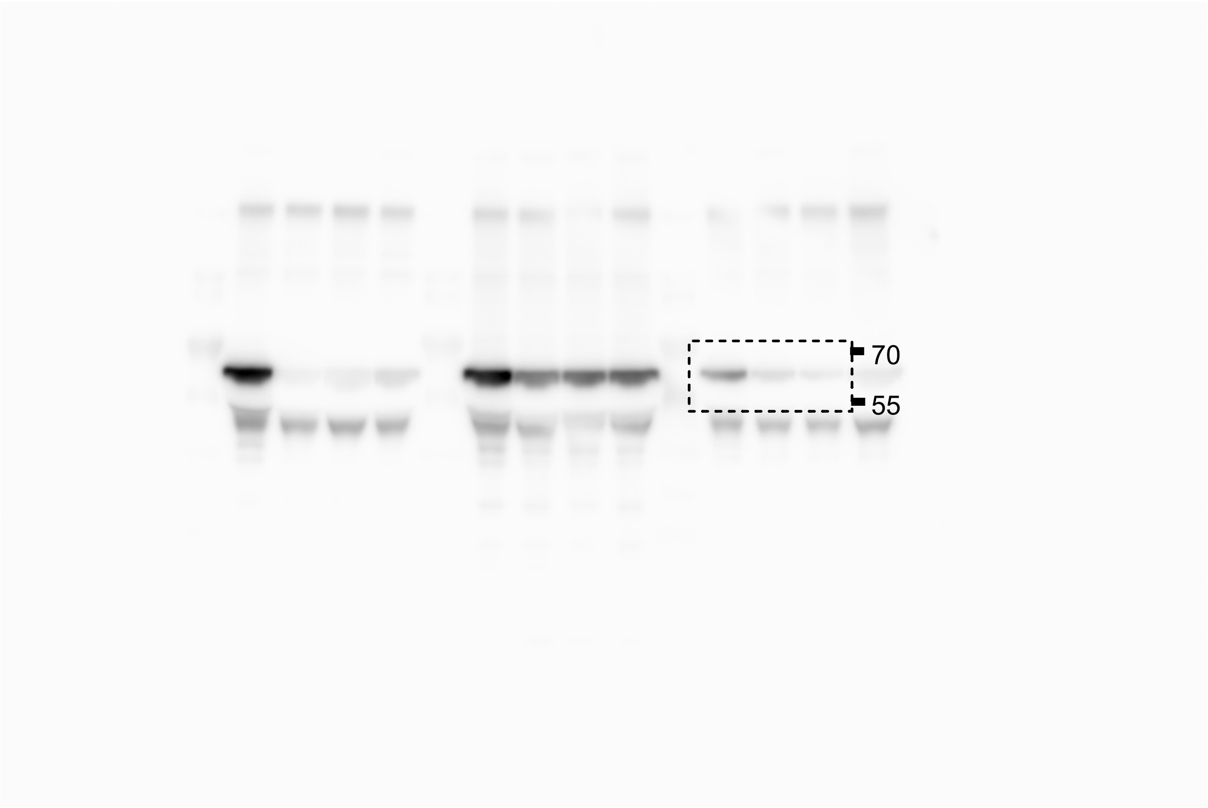

anti-β-actin

siControl siRELA#1 siRELA#2 (kDa)

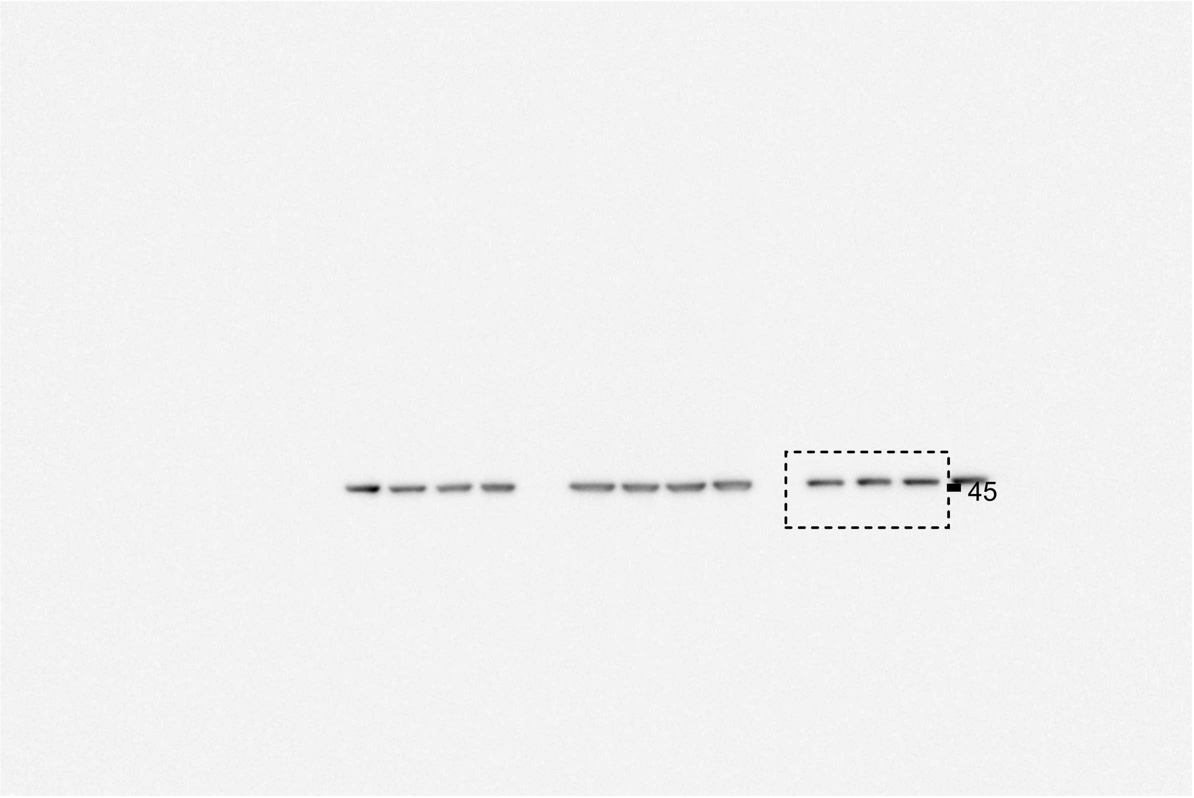

Supplemental Figure 2 Full-size images of western blotting

Fig. 2

D

MCC148
(lung SCC)

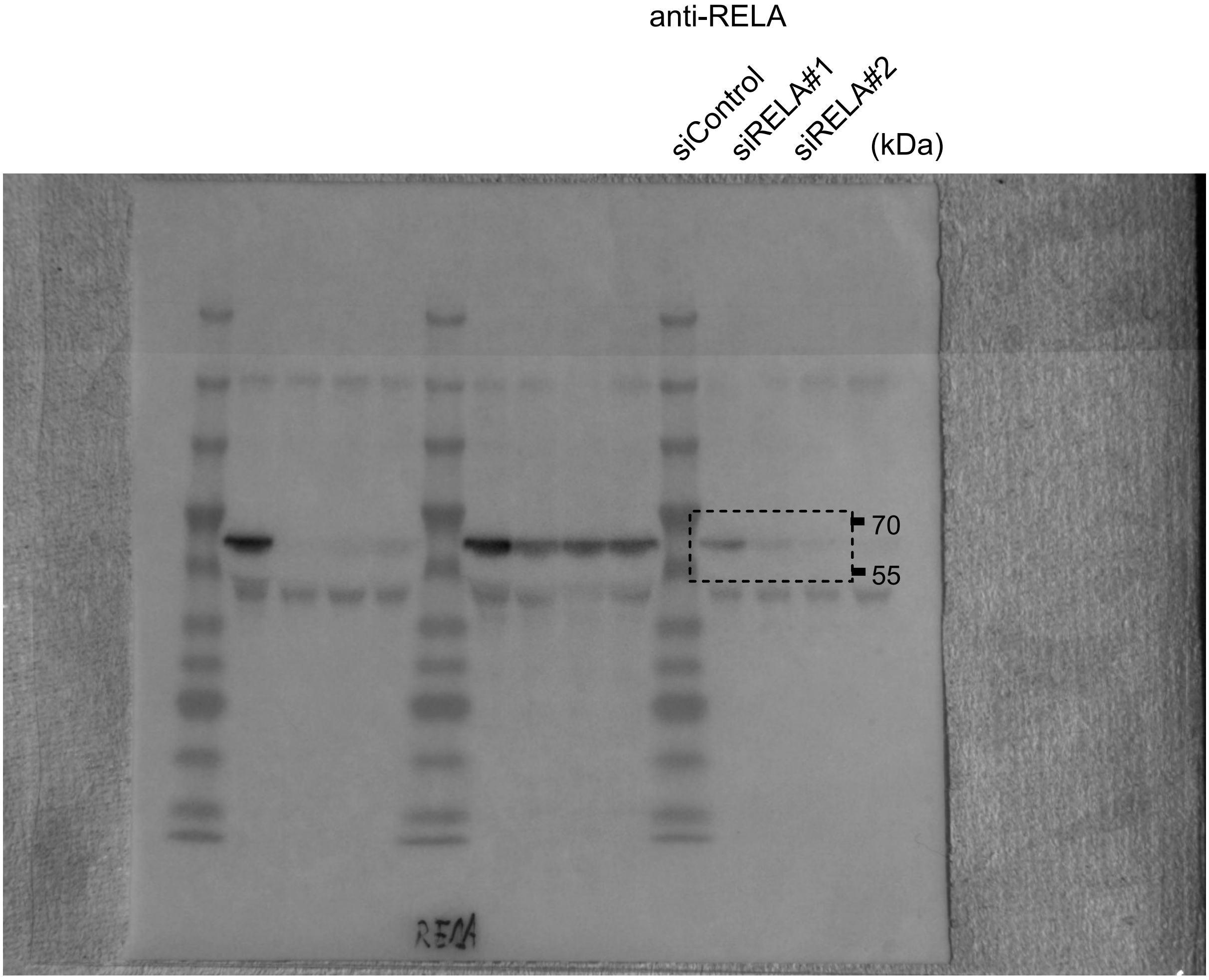

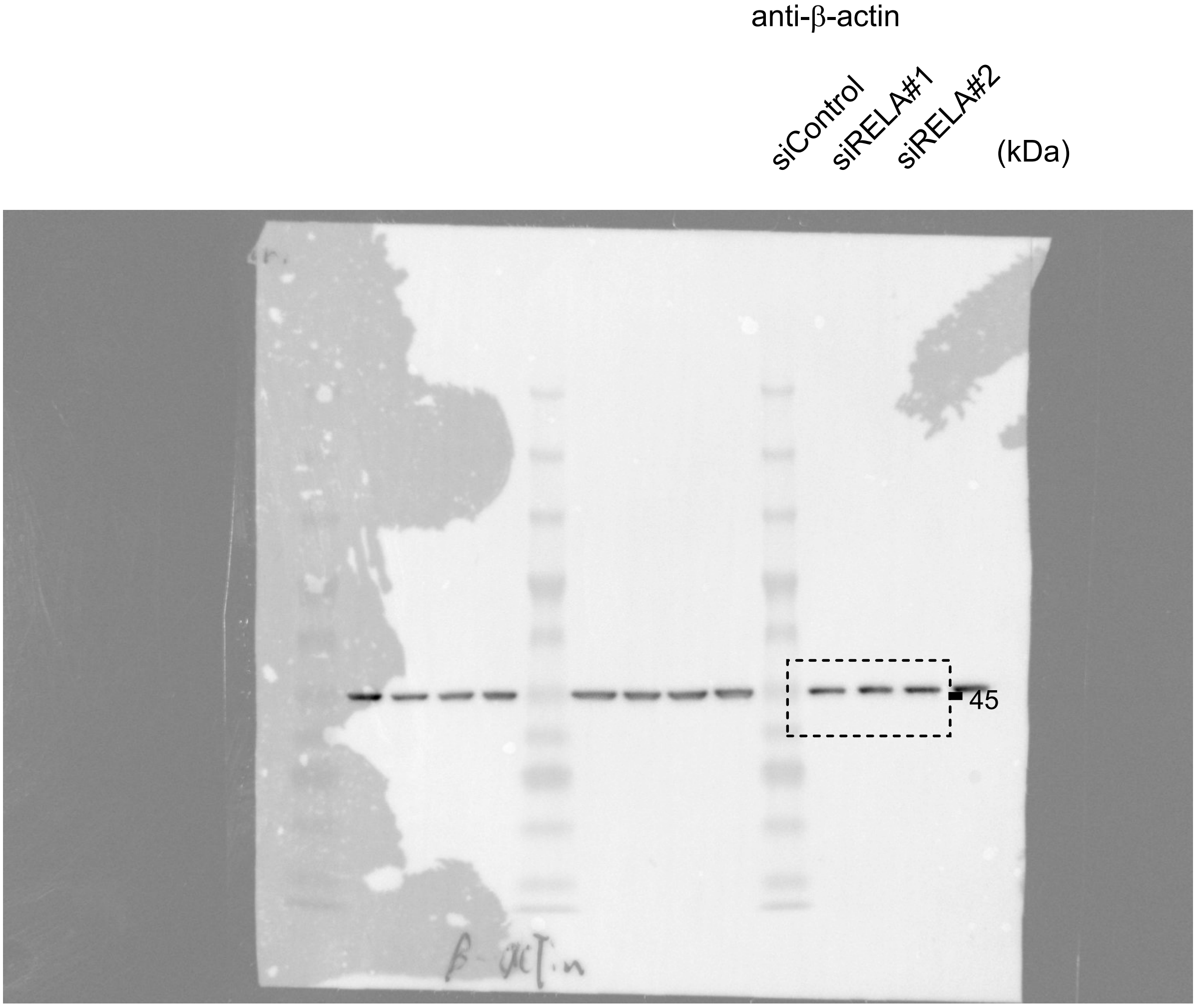

Supplemental Figure 2 Full-size images of western blotting

Fig. 2

E

Het1A
(normal)esphagus

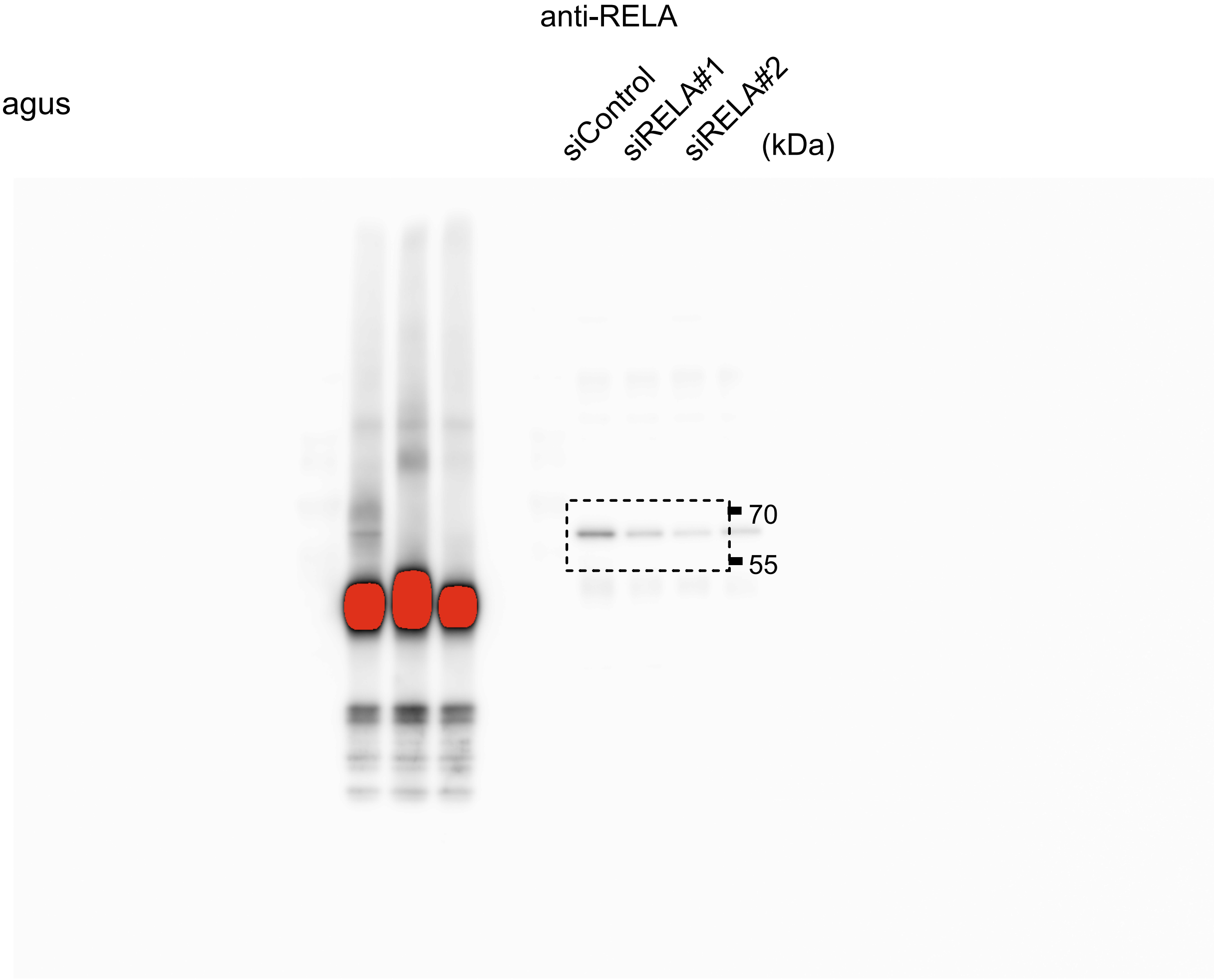

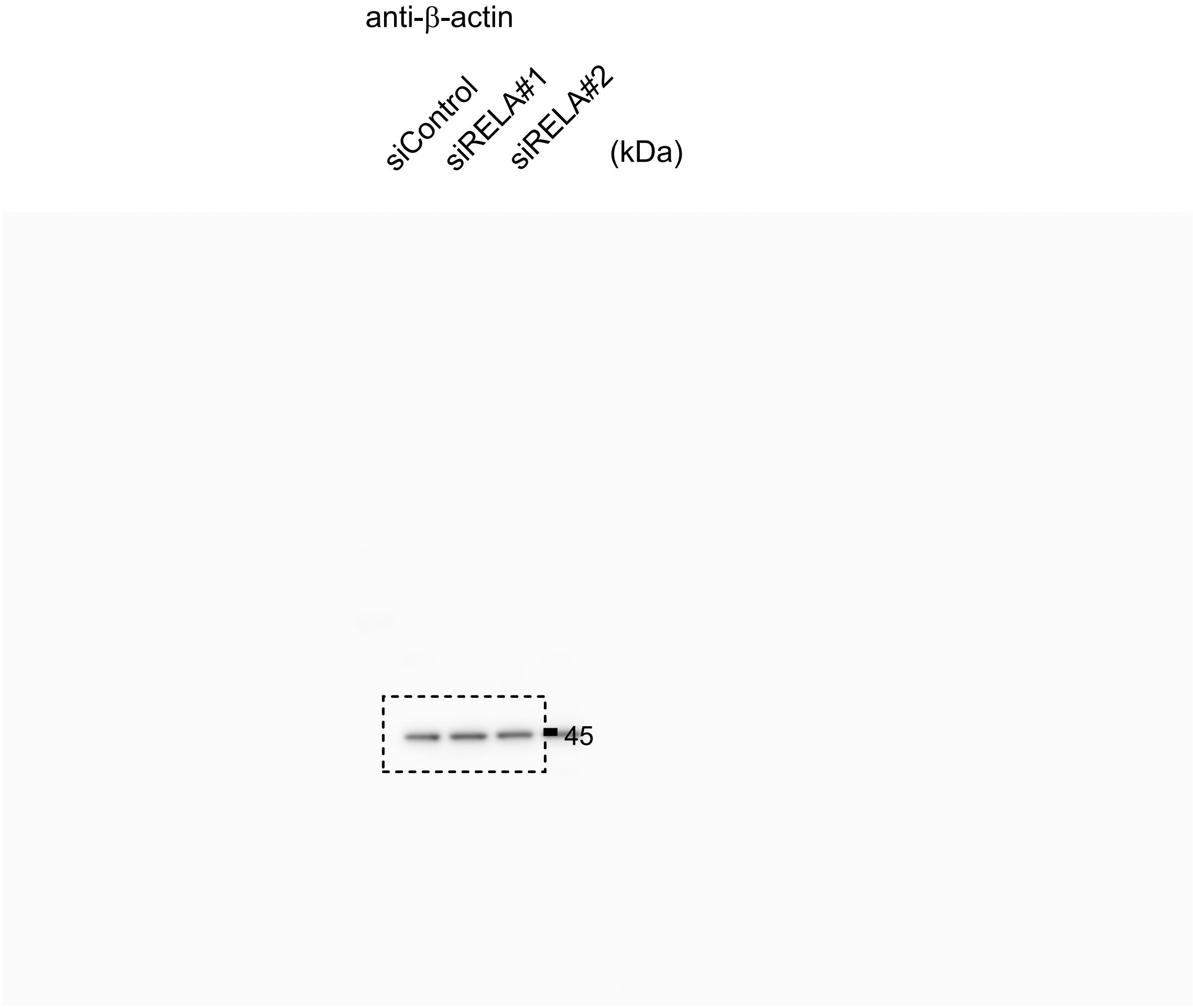

Supplemental Figure 2 Full-size images of western blotting

Fig. 2

E

Het1A
(normal)esphagus

anti-RELA

siControl siRELA#1 siRELA#2 (kDa)

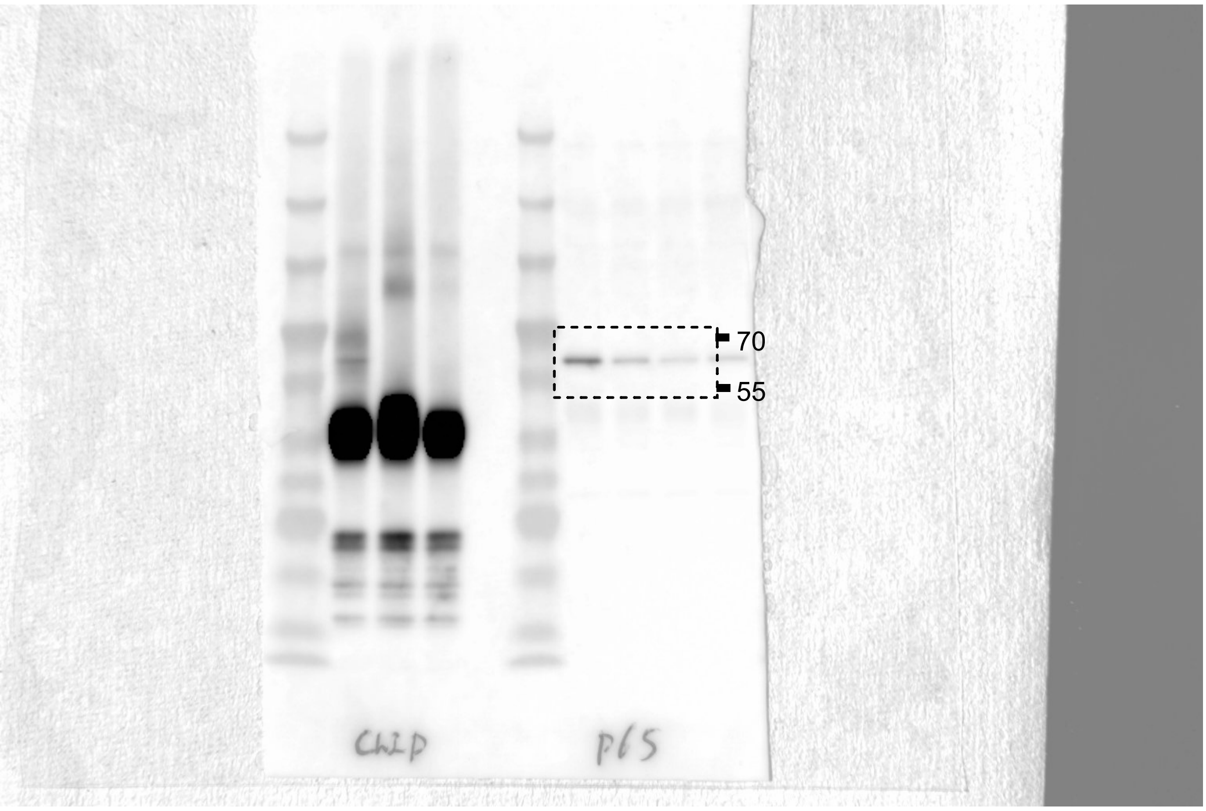

anti-β-actin

siControl siRELA#1 siRELA#2 (kDa)

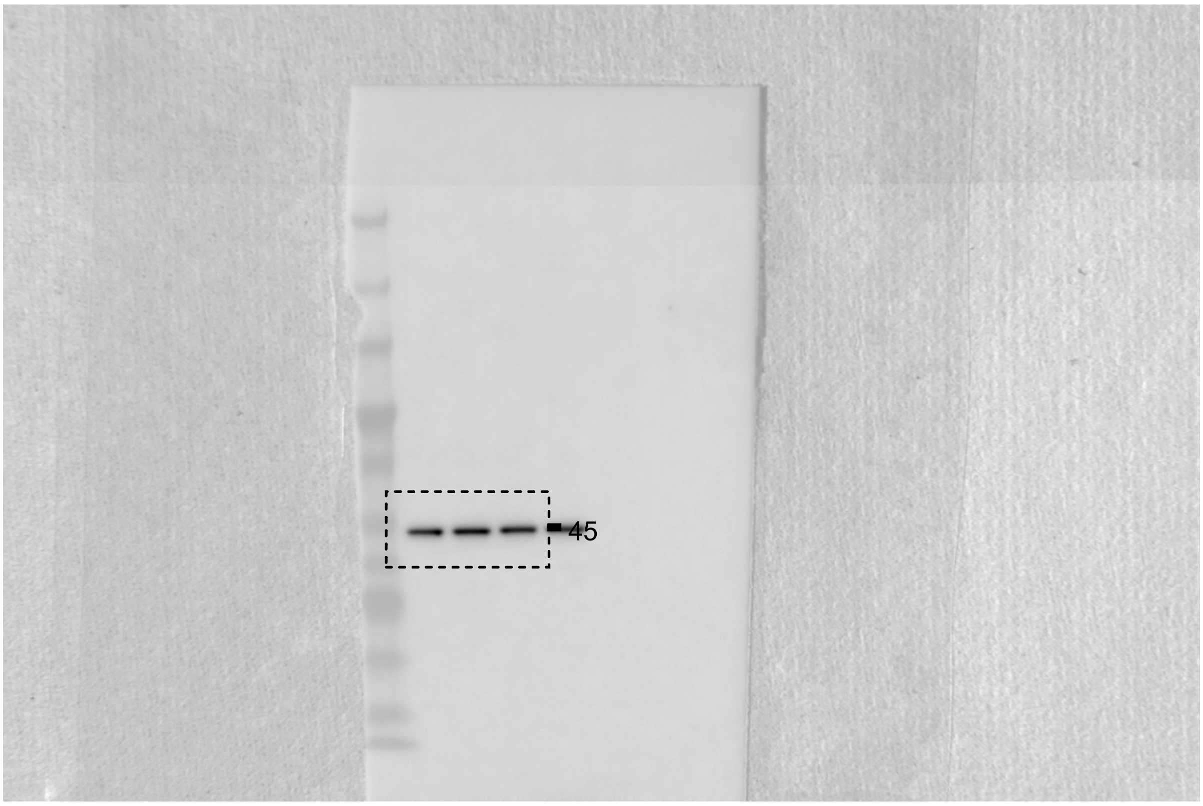

Supplemental Figure 2 Full-size images of western blotting

Fig. 2

F

IMR32
(neuroblastoma)

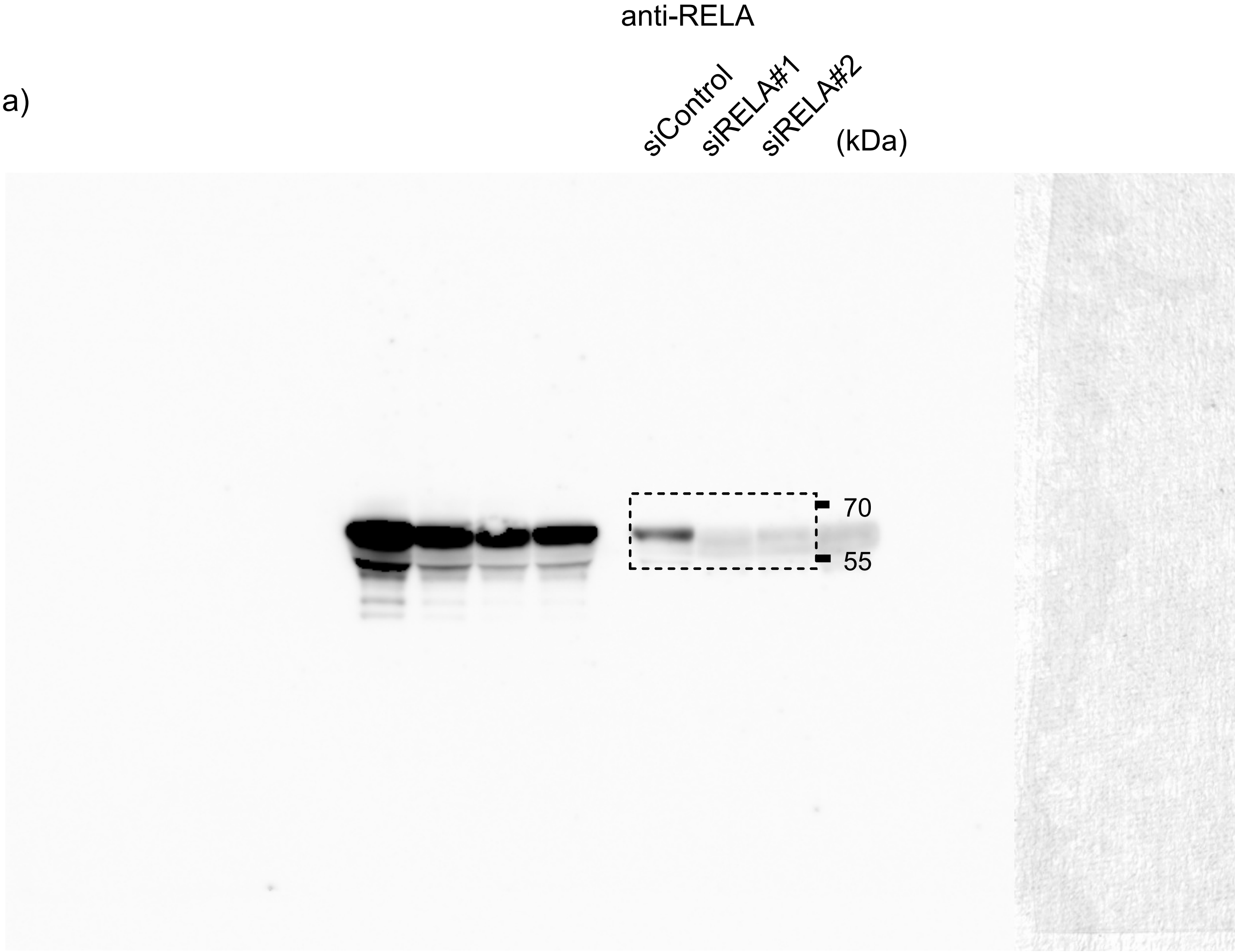

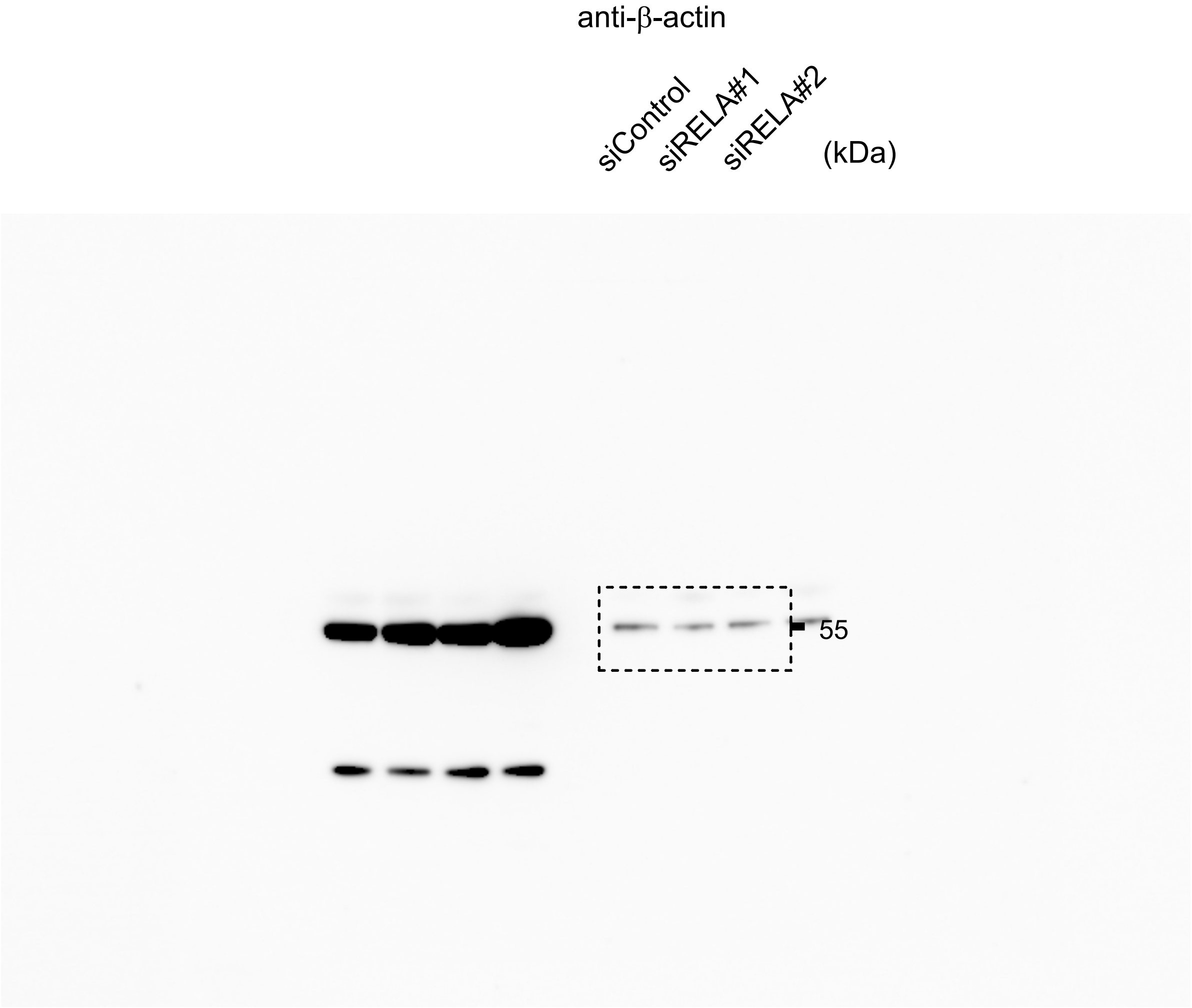

Supplemental Figure 2 Full-size images of western blotting

Fig. 2

F

IMR32
(neuroblastoma)

anti-RELA

siControl siRELA#1 siRELA#2 (kDa)

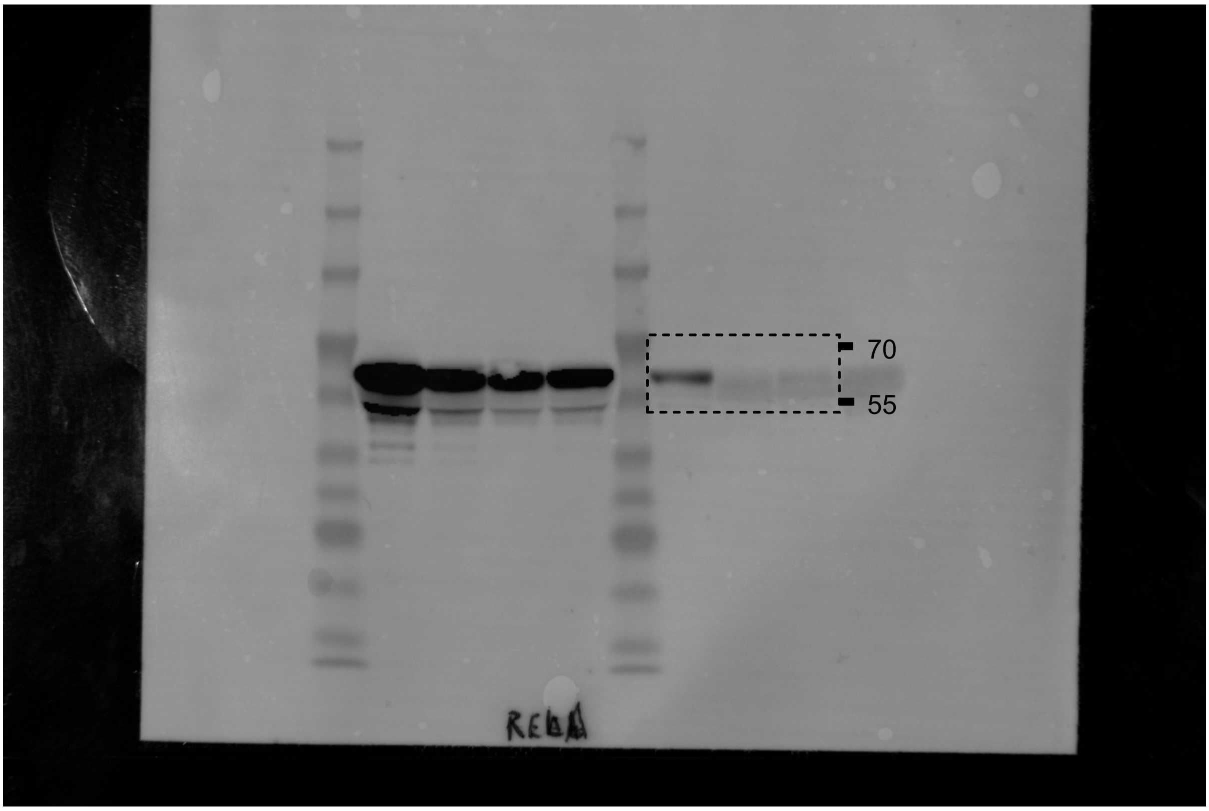

anti-β-actin

siControl siRELA#1 siRELA#2 (kDa)

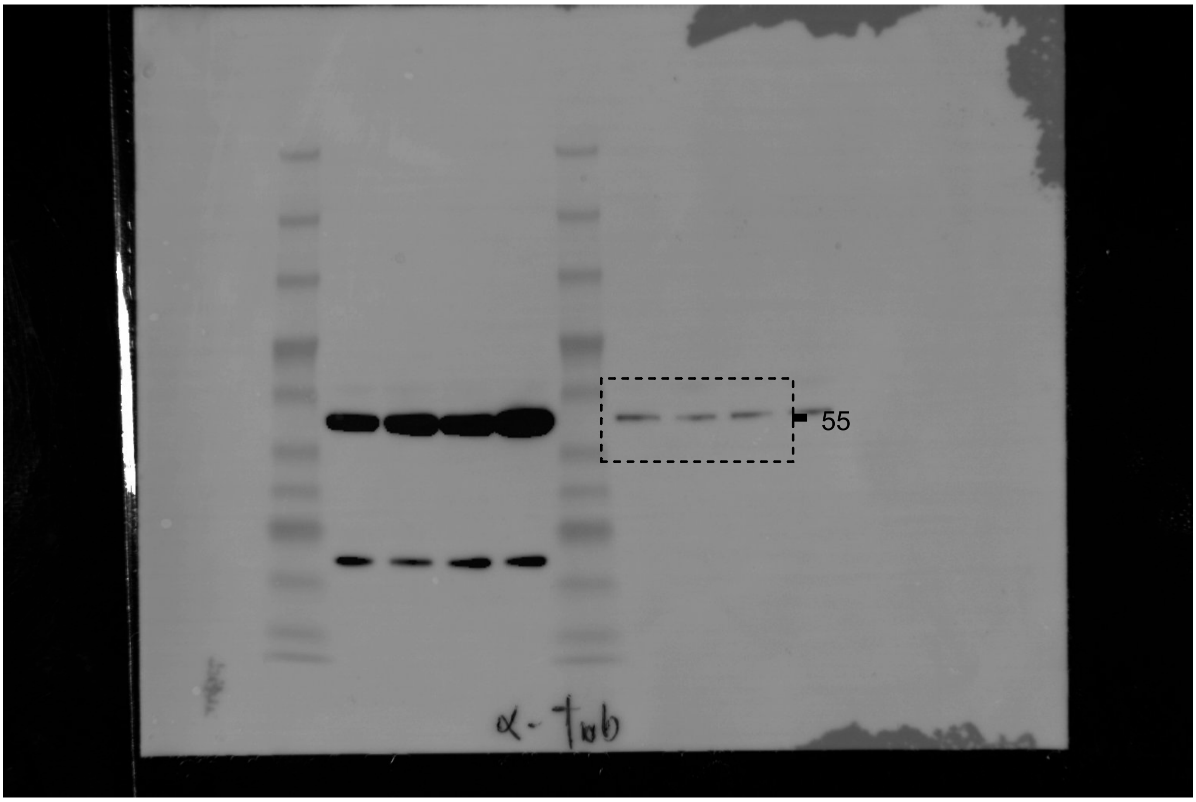

Supplemental Figure 2 Full-size images of western blotting

Fig. 3

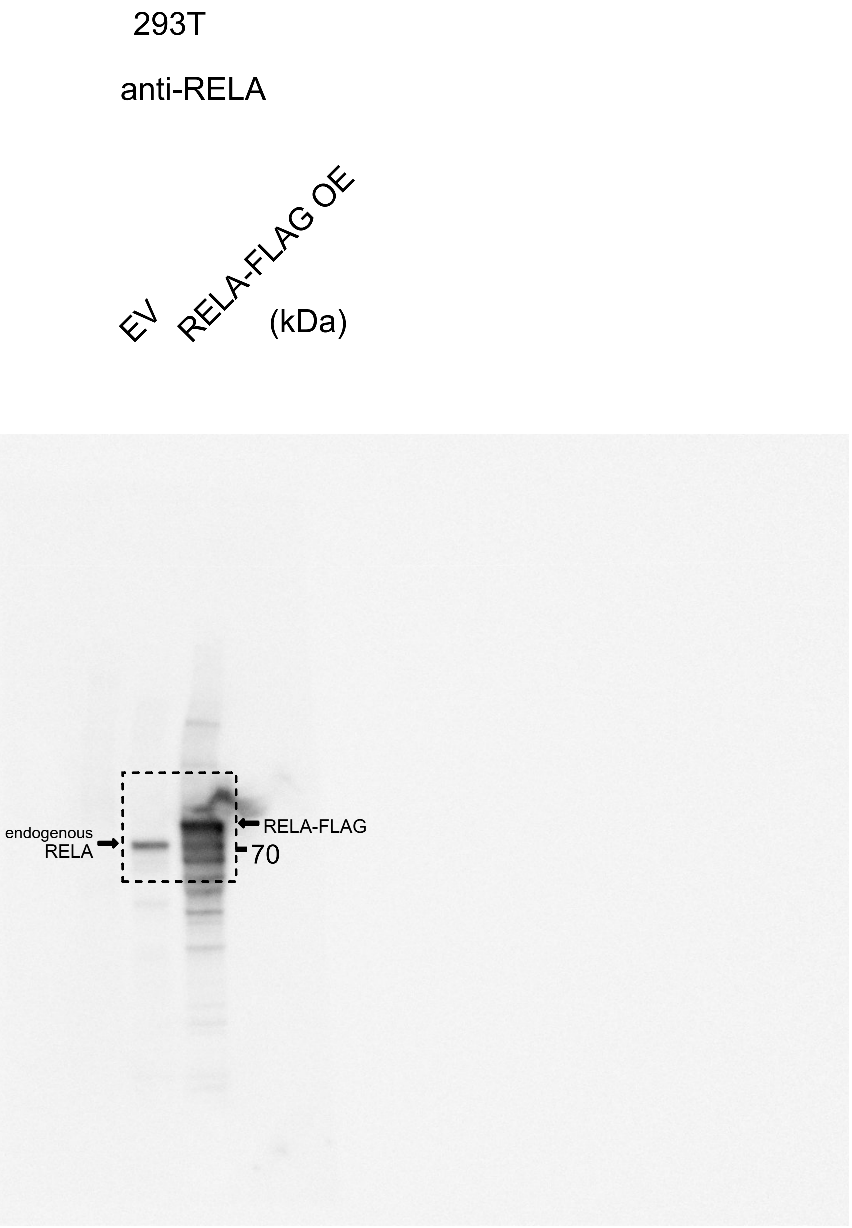

Supplemental Figure 2 Full-size images of western blotting

Fig. 3

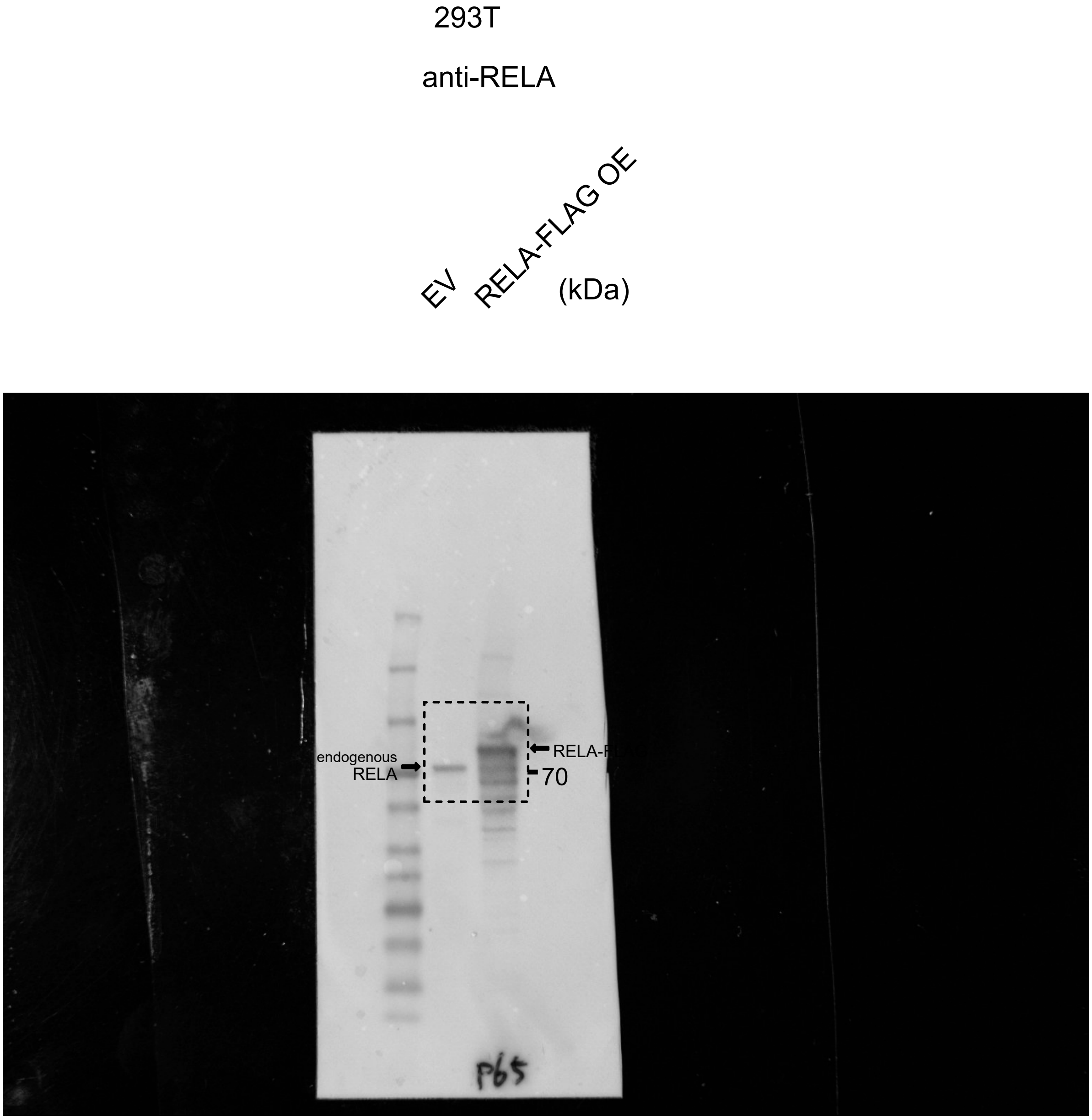

Supplemental Figure 2 Full-size images of western blotting

Fig. 3

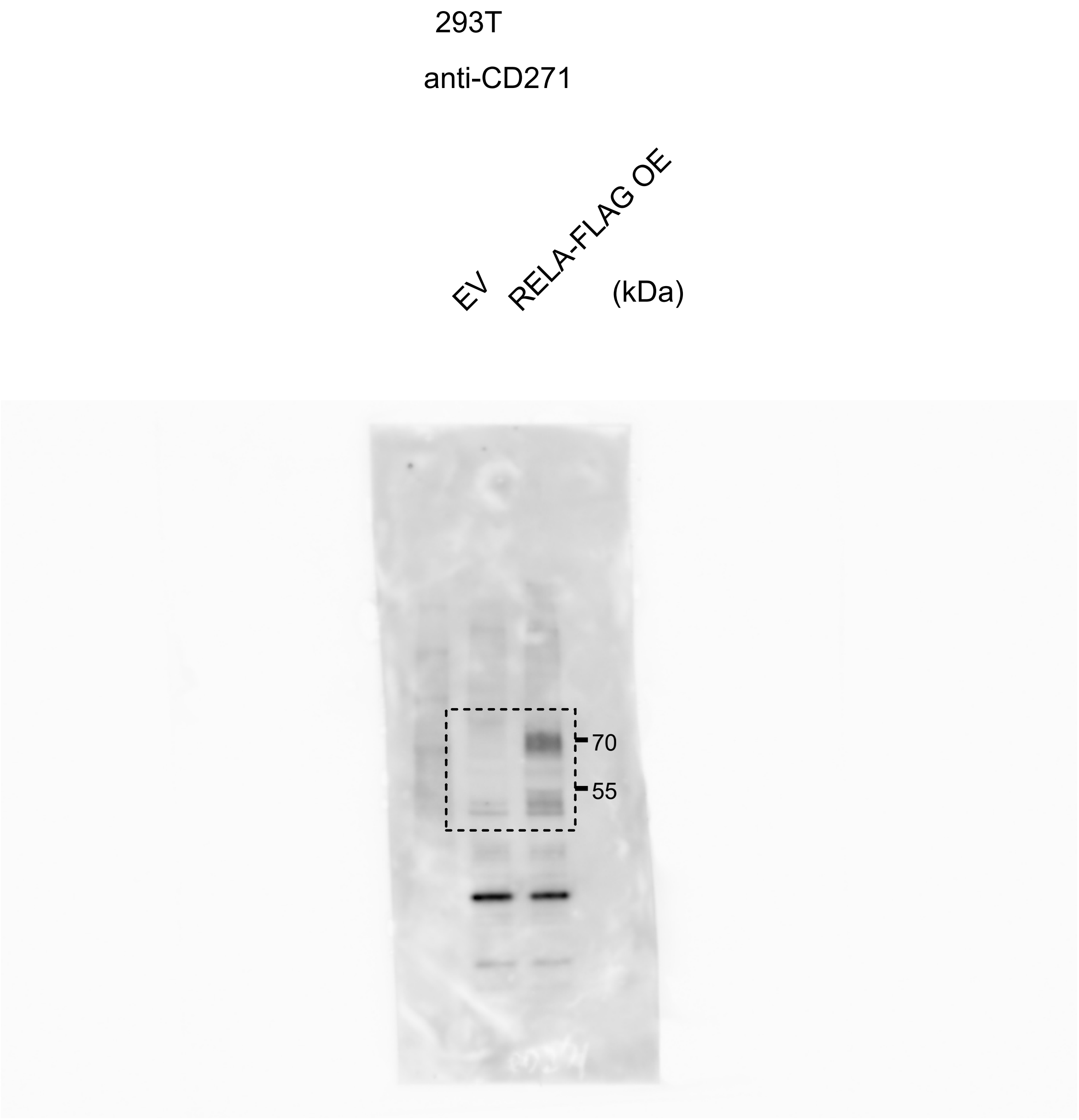

Supplemental Figure 2 Full-size images of western blotting

Fig. 3

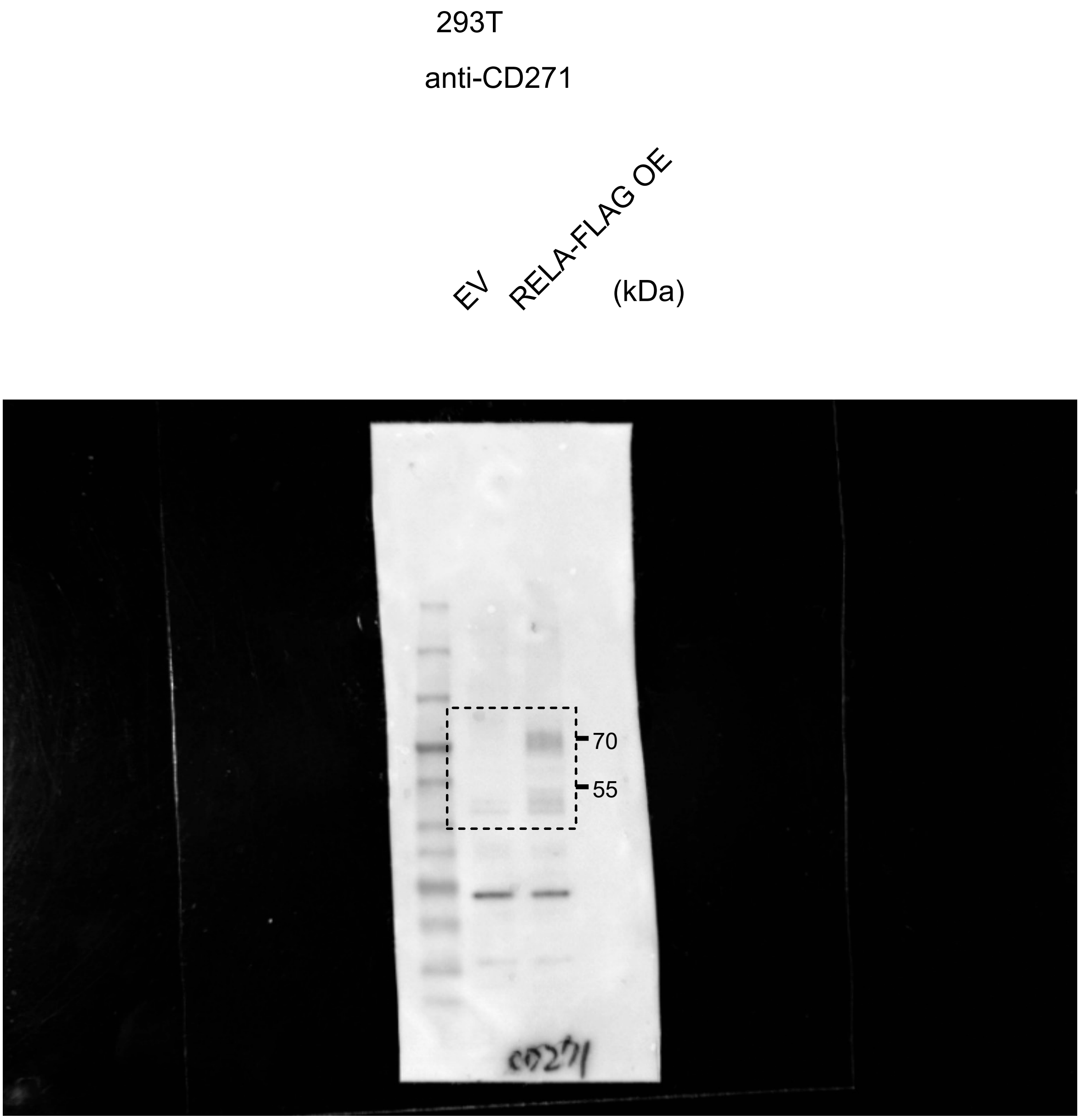

Supplemental Figure 2 Full-size images of western blotting

Fig. 3

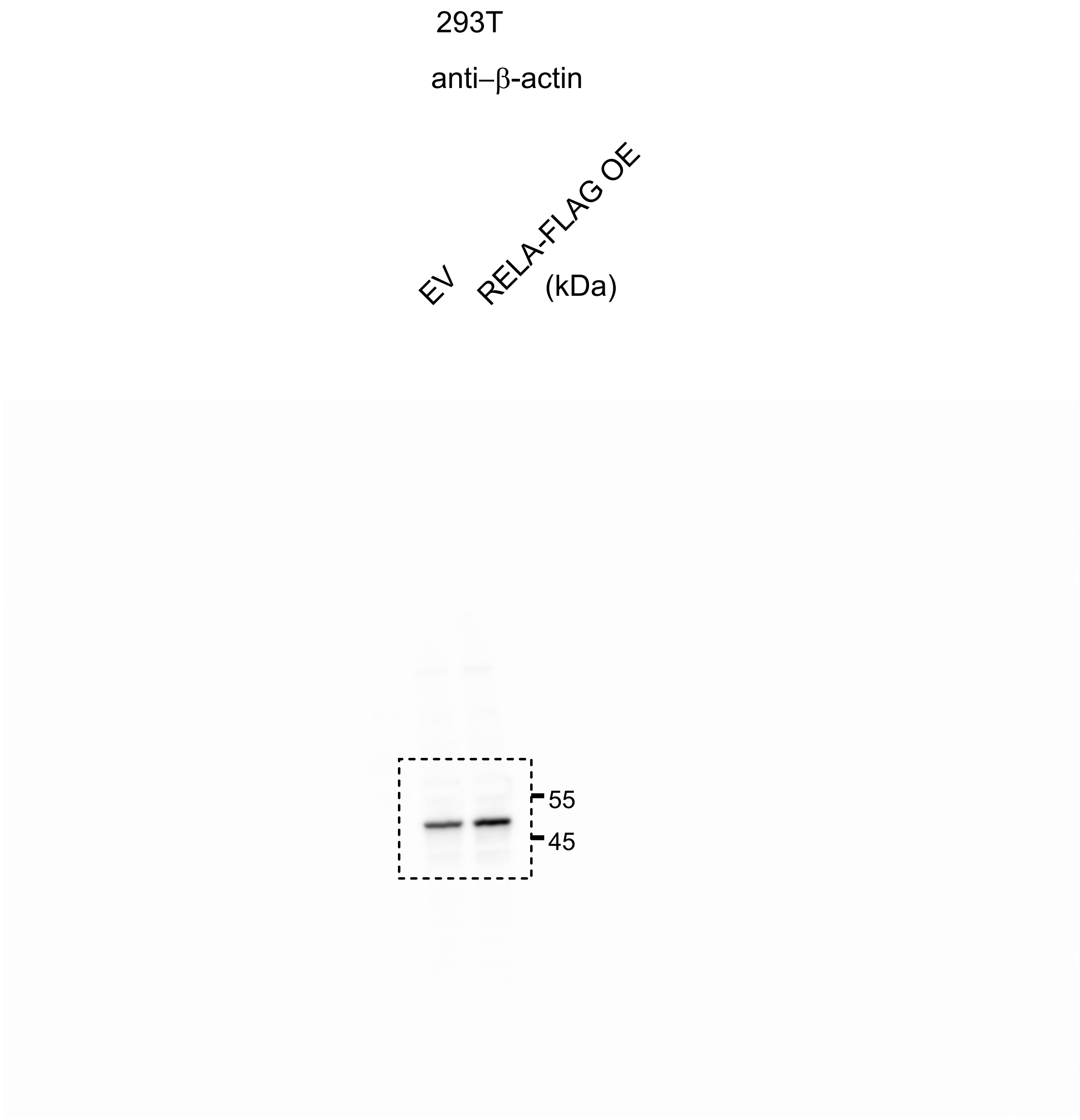

Supplemental Figure 2 Full-size images of western blotting

Fig. 3

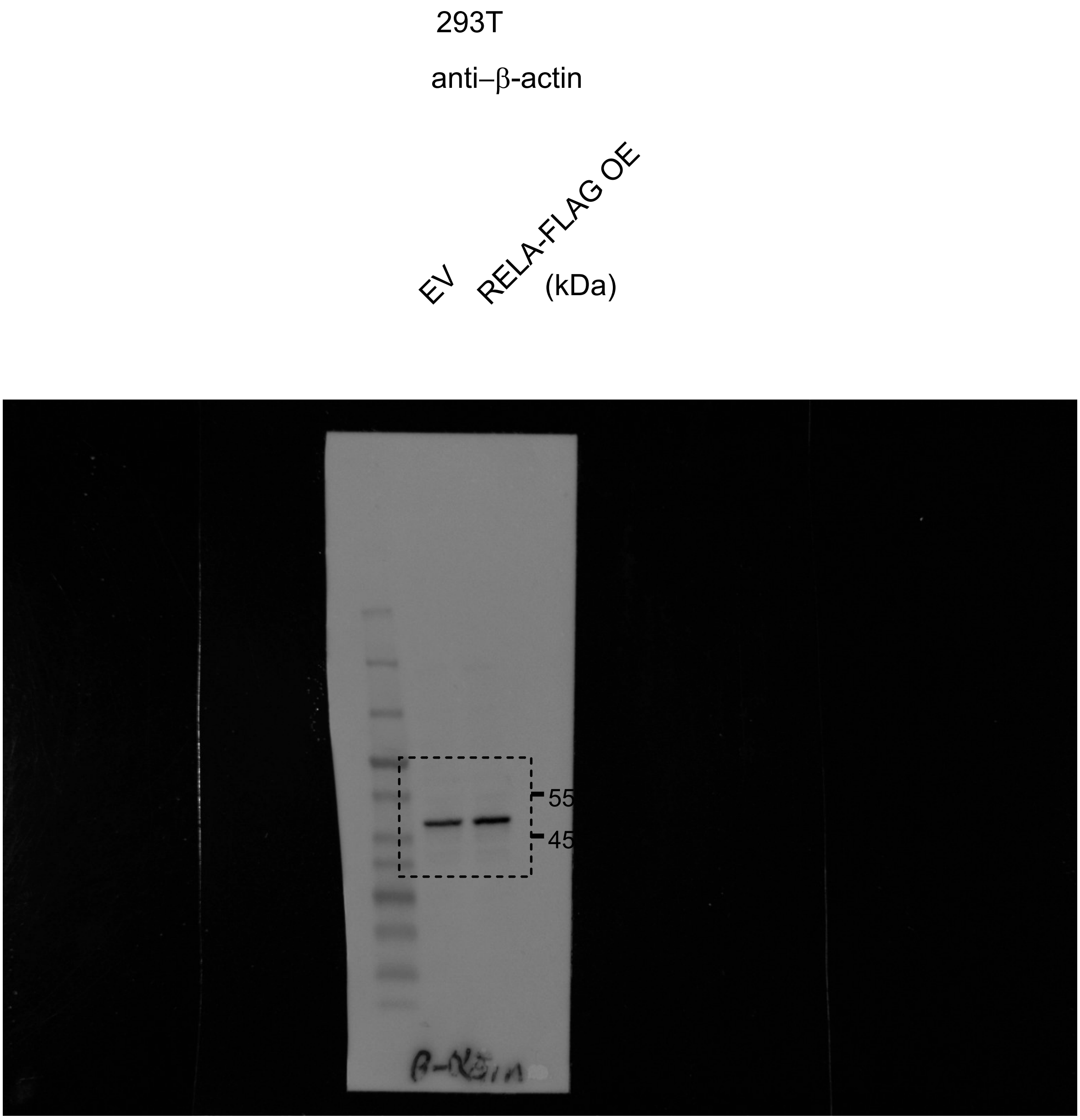

Supplement: Supplementary file 2 — Supplementary Figures. [file 41598_2022_22736_MOESM2_ESM.pdf]
